# Equivalent Scalar Stress Formulation Taking into Account Non-Resolved Turbulent Scales

**DOI:** 10.1007/s13239-021-00526-x

**Published:** 2021-03-05

**Authors:** Lucas Konnigk, Benjamin Torner, Martin Bruschewski, Sven Grundmann, Frank-Hendrik Wurm

**Affiliations:** 1 Institute of Turbomachinery, Faculty of Mechanical Engineering and Marine Technology, University of Rostock, Rostock, Germany; 2 Institute of Fluid Mechanics, Faculty of Mechanical Engineering and Marine Technology, University of Rostock, Rostock, Germany

**Keywords:** Blood damage, Computational fluid dynamics, Direct numerical simulation, Dissipation, Shear stresses, Turbulence

## Abstract

**Purpose:**

Cardiovascular engineering includes flows with fluid-dynamical stresses as a parameter of interest. Mechanical stresses are high-risk factors for blood damage and can be assessed by computational fluid dynamics. By now, it is not described how to calculate an adequate scalar stress out of turbulent flow regimes when the whole share of turbulence is not resolved by the simulation method and how this impacts the stress calculation.

**Methods:**

We conducted direct numerical simulations (DNS) of test cases (a turbulent channel flow and the FDA nozzle) in order to access all scales of flow movement. After validation of both DNS with literature und experimental data using magnetic resonance imaging, the mechanical stress is calculated as a baseline. Afterwards, same flows are calculated using state-of-the-art turbulence models. The stresses are computed for every result using our definition of an equivalent scalar stress, which includes the influence from respective turbulence model, by using the parameter dissipation. Afterwards, the results are compared with the baseline data.

**Results:**

The results show a good agreement regarding the computed stress. Even when no turbulence is resolved by the simulation method, the results agree well with DNS data. When the influence of non-resolved motion is neglected in the stress calculation, it is underpredicted in all cases.

**Conclusion:**

With the used scalar stress formulation, it is possible to include information about the turbulence of the flow into the mechanical stress calculation even when the used simulation method does not resolve any turbulence.

## Introduction

Computational fluid dynamics (CFD) is a major branch in the field of numerical methods in biomedical engineering. CFD can provide detailed insights into flow systems of the human body. In many cases, velocities within these systems are low and/or the vessel diameters are sufficiently small, so that the friction forces in the fluid are dominant over the inertial forces. Therefore, the flow can be assumed to be laminar and the accuracy of CFD mostly depends on the mesh quality and on the accuracy of the boundary conditions. Not exclusively, but for particular cases and devices of biomedical engineering, the blood flow is artificially modified and the flow transitions from laminar to an intermediate or even fully turbulent state.^36^ Ventricular assist devices (VADs) are an example of such complex intervention.^13^,^48^ As a result, the spatial and temporal progression of local fluid properties, like velocity and pressure, becomes increasingly turbulent, therefore time-dependent, and three-dimensional.

In a fully turbulent flow, the kinetic energy budget of turbulent motion can be described by the energy cascade.^23^ This theory describes the evolution of turbulence from its origin on large, energy-rich and anisotropic scales (energy containing range) followed by the breakup of large structures into smaller structures with continuously decreasing length and time scales (inertial subrange). At the smallest scales, the turbulent motion is isotropic and contains less energy. The vortices do not break up any further, but their kinetic energy is converted into internal energy through dissipation (viscous subrange). A direct numerical simulation (DNS) is necessary in order to be able to capture all scales mentioned above in a numerical prediction of the turbulent flow.^45^ A sufficiently fine mesh and small enough time steps are necessary to resolve the smallest structures of the turbulent motion. It is obvious, that conducting a DNS can be very time-consuming, demands much computing power and is simply not possible for many engineering applications yet. Hence, approaches for modeling the turbulence are useful and effective. Turbulence models based on the Reynolds-averaged Navier-Stokes (RANS) equations are the most-used modeling approaches for industrial applications. They all have in common that they only account for the turbulence on a statistical approach without resolving turbulent motion. An important approach for turbomachines is the unsteady-RANS (URANS). Here, the time dependent fluid motion is considered in the governing equations in a kind of low pass filter concept, where the turbulent motion is still modeled. RANS and URANS models are known to be relatively dissipative,^39^ which means that they possibly diminish velocity fluctuations even for a time-dependent simulation.

Another approach is the use of a large eddy simulation (LES). Unlike RANS, a spatial averaging is applied where small turbulent structures still need to be modeled but larger turbulent structures are resolved by the grid. A much higher resolution than for RANS models is required but the influence on the flow is substantially better simulated than with RANS methods. Note that because of the mentioned isotropy of the small structures and their lower energy content, their influence is easier to model than the entire scales of turbulent structures. The computational effort lies between RANS and DNS. The mesh does not require to be as fine as for a DNS, but the resolution of the near-wall layer becomes crucial with increasing Reynolds number. Since the majority of turbulent kinetic energy (TKE) is produced in the vicinity of the walls, many grid nodes are needed here to reflect the physics properly. Because of this requirement, an adequate wall-resolving LES of a wall-bounded flow is sometimes called *quasi-DNS*.^28^ Hybrid methods between LES and RANS can be a promising compromise between efficiency and accuracy but are not covered in this study.

**Figure 1:**
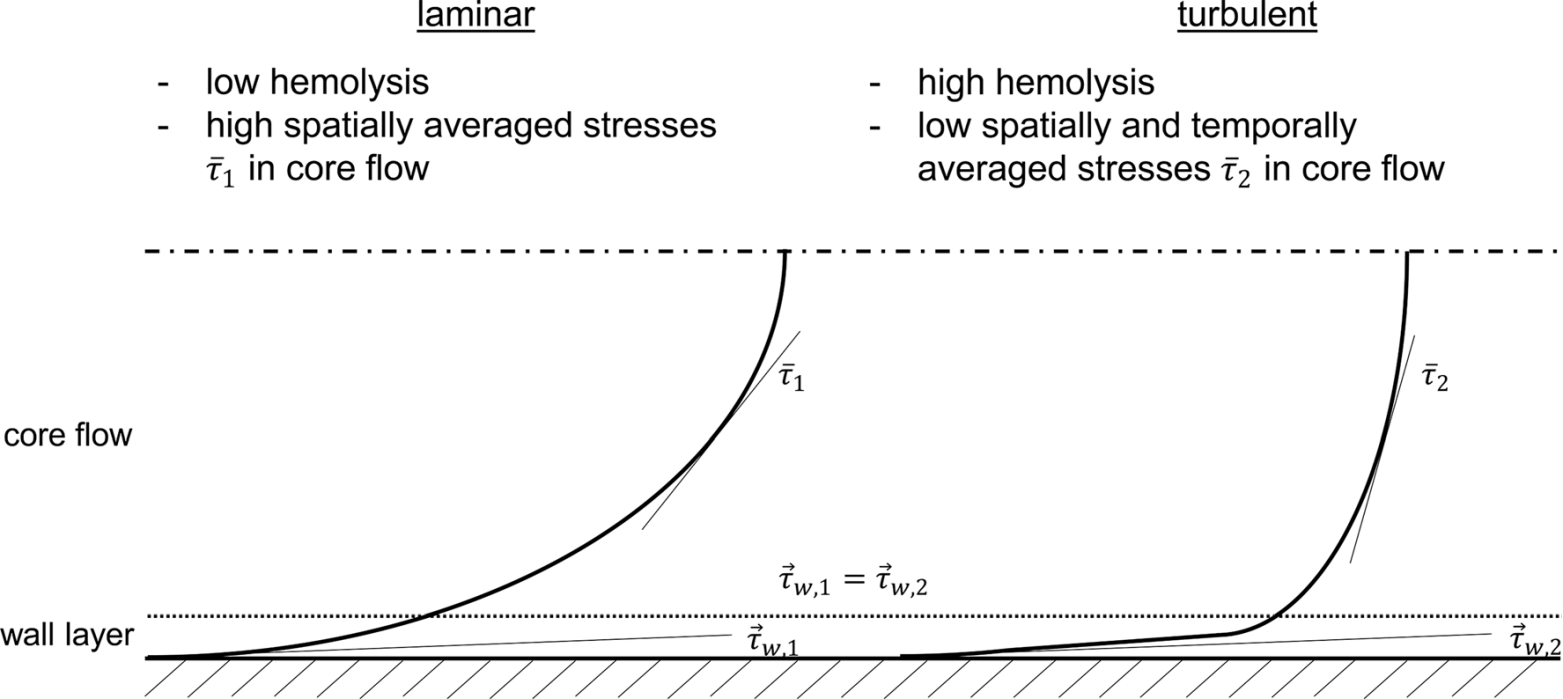
Schematic representation of two mean velocity profiles in laminar and turbulent flow with equal mean wall shear stress $\vec{\tau }_{w}$ but different average stresses $\bar{\tau }$ in the core flow to explain the statements in the study by Quinlan.^46^

VADs are mechanical assist devices for patients who suffer from terminal heart failure and are not eligible to wait for a donor heart. Our previous work has focused on the influence of turbulence in VADs,^55^ on the verification of VAD flow simulations with RANS and LES,^34^,^35^ and on the comparison of results between simulation methods with different influence of turbulence modeling.^54^ Turbulence models based on the RANS equations are the most used models in VAD research.^13^ For blood flow, it is commonly accepted that turbulence has an impact on the cells within the plasma.^1^,^44^,^58^ Generally speaking, velocity gradients in the flow field and therefore mechanical normal and shear stresses, hereinafter just shear stresses or stresses, can cause blood damage. The term (flow-induced) blood damage includes alteration of proteins (van Willebrand factor, vWF), activation of platelets, and damage to red blood cells (RBCs), due to the high stresses which could lead to adverse events for the patient like gastrointestinal bleeding, thrombus formation and stroke, or hemolysis, respectively.^19^,^52^ Numerous experiments have been done to quantify the influence of turbulence to blood damage.^2^,^12^,^27^,^47^,^51^ Kameneva *et al.*^31^ carried out pipe flow experiments with an RBC-loaded fluid under different laminar and turbulent conditions ${\text{Re}} = 300 \ldots 5100$. They used the dependency of pressure drop and wall shear in a pipe, in combination with a varying fluid viscosity, to study the hemolysis between flows of same wall shear but under either laminar or turbulent flow state. They showed that the RBCs are more damaged under turbulent flow in comparison to the laminar pipe flow with identical pressure drop, i.e. wall shear. Quinlan^46^ used the experimental data from Kameneva *et al.* for further analysis. He argued that an equal wall shear stress is not equivalent to equal stresses in the flow field. He further explained that the stresses in the spatial mean in a laminar flow with a parabolic velocity profile, are two thirds of the wall shear stress. For the turbulent case, he estimated the spatial and temporal mean of the stresses based on the viscous wall layer with a linear velocity profile, patched to a log-law profile for a temporally mean velocity across the pipe cross section. He calculated that the ratio of wall shear stresses to stresses in the flow field is smaller, namely between 0.27 and 0.14 for the corresponding Reynolds numbers of Kameneva *et al.*, and thus smaller than for the laminar case. The statements of Quinlan can be simplified by a schematic representation Fig. 1. He concluded, that even with a higher hemolysis for turbulent cases, the (time-averaged) shear stresses of the time-averaged turbulent field were five times smaller than the overall stresses from comparable laminar cases. This observation motivates the definition of an additional turbulent mechanical stress variable $\tau_{\text{turb}}$ added to the direct mechanical stress $\tau_{\text{dir}}$ (often called $\tau_{\text{mean}}$ but was renamed since we link it to the variable direct dissipation $\varepsilon_{\text{dir}}$), which is calculated from the time-averaged velocity gradients, to access the influence of turbulence to the overall, i.e. total, acting mechanical stress $\tau_{\text{tot}} = \tau_{\text{dir}} + \tau_{\text{turb}}$ in the flow field. This is especially necessary for RANS results, because in this case only the temporal averaged flow field is available for stress calculation. Note that $\tau_{\text{turb}}$ is not to be confused with the Reynolds stresses that need to be modeled in turbulence modeling. Instead, $\tau_{\text{turb}}$ is intended to describe the influence of turbulence, i.e. gradients of the fluctuating velocity components, on blood components in the form of a mechanical stress. Inversely, this means that an estimation of the blood damage from a CFD of a turbulent flow is most likely underestimated if only the time-averaged stresses are used. Our previous as well as other comprehensive sensitivity analyses showed that an increasing resolution of turbulence in the flow leads to an increasing prediction of blood damage.^21^,^34^ Since it is still not practicable to include the complete turbulent field in the CFD analysis (by DNS) to calculate the blood damage, methods have to be established to include the unresolved part of the turbulence statistically in the prediction.

First experimental studies tried to link Reynolds stresses with $\tau_{\text{turb}}$^12^,^47^ and a correlation with blood damage could be found. However, further work revealed that Reynolds stresses cannot describe $\tau_{\text{turb}}$ and the resulting blood damage adequately^16^,^20^,^25^,^30^,^46^ since they rather constitute a momentum transfer due to turbulent movement than a mechanical stress. Therefore, a correlation between Reynolds stresses and blood damage exists since blood damage increases with increasing turbulence in the flow. However, Hund *et al.*^26^ showed that this dependency is nonlinear.

Another approach to fill the gap between $\tau_{\text{dir}}$ and $\tau_{\text{tot}}$ acting on the blood cells is to utilize the energy dissipation and to link it with blood damage.^16^,^26^,^30^,^41^,^46^ As already mentioned when describing the energy cascade, fluid dynamic dissipation describes the conversion of kinetic energy into heat due to friction, i.e. mechanical stress/velocity gradients. Early experiments from Bluestein and Mockros^3^ found that the average rate of hemolysis correlates with the average dissipation rate. Hund *et al.*^26^ also showed that dissipation is a more appropriate metric for hemolysis than the Reynolds stresses. However, the link between blood damage and dissipation is not straight forward. In turbulent flows, energy dissipation, i.e. power loss for the flow, occurs over the whole spatial and temporal range of motions and as a common procedure for turbulent flows, it can be statistically separated into mean/direct and turbulent part. First, turbulent dissipation occurs at the smallest and fastest scales (Kolmogorov scale) in the flow.^45^ Secondly it also occurs on the largest and slowest scales: the mean motion, e.g. near-wall gradients. It is called direct dissipation, because it does not follow the energy transfer from the large scales to the Kolmogorov scales, in the context of the theory of the turbulent energy cascade.^24^ In other words, direct dissipation corresponds to the statistical energy loss due to time-average velocity gradients in order to separate this part from the energy loss due to turbulence alone. For laminar flows, direct dissipation is the only reason for blood damage.

Wu *et al.*^57^ correlated overall dissipation with hemolysis in the capillary tube experiments from Kameneva *et al.* They found a direct dependency between damaged red blood cells and dissipation rate, regardless of laminar or turbulent flow state. This further indicates that dissipation could be the parameter that includes the additional influence of turbulence on the blood damage. In Reference 57 the dissipation was computed from the experimental pressure drop and flow rate, which is the integral flow loss *via* the whole domain.

To conclude the most important findings of latest research: (1) dissipation as a physical quantity is directly related to blood damage, whereat hemolysis was the most studied damage type and (2) fluid dynamic shear stress, as an accepted parameter of flow-induced blood damage, can be expressed with energy dissipation.

In this study we present a new approach to determine a scalar equivalent stress based on the dissipation without the need to resolve all turbulent scales but with the stress information of the un-resolved scales included. This is especially important for CFD in VADs, because those flow fields are commonly simulated by RANS or, in few cases only, using LES methods. Furthermore, with this study we want to analyze how a calculation of mechanical stresses differs depending on whether the modeled field is included in the calculations or not. This study is therefore limited to the application of a scalar equivalent stress to perform a fast but more reliable numerical evaluation of different designs or related issues.

We carried out DNS computations of two test cases to examine our hypothesis. The cases are a turbulent channel flow and the FDA (U.S. Food and Drug Administration) case “nozzle with sudden expansion”.^50^ The flow characteristics of chosen cases, i.e. wall bounded flows, high velocity gradients, or pressure gradients, are transferable to a VAD flow. We compared our results with literature DNS data (channel) and with experimental results (nozzle) for validation. Experimental values for validation were the same as Stewart *et al.*^50^ (officially used by the FDA).

Furthermore, we performed additional velocity field measurements in the nozzle to increase the data base for validation. In this study, we chose magnetic resonance imaging (MRI), to provide a reference data set for the mean flow field. The purpose of the MRI velocity measurements is to validate the numerical model in the entire flow domain, in particular the boundary conditions of the DNS, and increase the confidence in the numerical approach.

After successful validation, we simulated same flows with state-of-the-art turbulence modeling methods. In the final step, we computed the stress field out of all generated flow fields using our scalar equivalent stress on the one hand and with existing methods from the literature on the other hand. These results were then analyzed with respect to their differences in order to assess the effect of the unresolved scales.

## Methods

### Computational Setups

Various numerical simulations were carried out for this study. Before the details of the respective setups are presented, the commonalities of the individual setups will be mentioned here. For both geometries results of a DNS, an LES and RANS simulations are presented. Solving of the flow equations, as well as the pre- and postprocessing was performed with the commercial software ANSYS CFX 18.0 (ANSYS, Inc., Canonsburg, USA) and the open-source software ParaView 5.9.0.^22^ The hexahedral, block-structured meshes were created for all geometries with ANSYS ICEM CFD 18.0. Simulations were performed on a high-performance computing cluster in double precision mode.

#### Turbulent Channel Flow

The turbulent channel flow was computed at a friction Reynolds number ${\text{Re}}_{\tau } = u_{\tau } h/\nu$ of 180 including the channel half-height $h$. The channel width is $2\pi h$ and its length is $5\pi h$. It could be shown by Kim *et al.*^33^ using two-point correlations that the domain size is adequate. With prescribed wall shear stress $\tau_{w}$, a corresponding pressure drop can be calculated and used as a boundary condition between inlet and outlet. A no-slip condition was set at the hydraulically smooth upper and bottom walls. Besides that, all other boundaries, including inlet and outlet, were implemented as periodic boundary conditions. During post-processing, *x*-*z*-planes were set at various *y*-positions and the area-average of all variables of interest were obtained at these positions to receive two-dimensional results of the time-averaged flow field.

#### Channel: Direct Numerical Simulation

**Figure 2:**
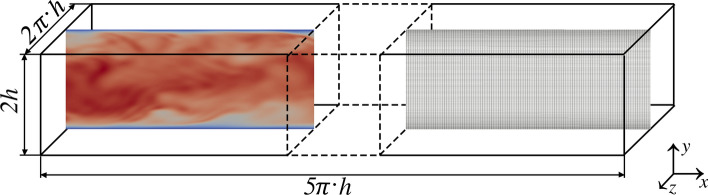
Instantaneous velocity magnitude where red symbolizes a high and blue means a low velocity, cutout from the DNS mesh, as well as dimensions of the computational domain.

The mesh size is selected such that all turbulent scales essential for the primary flow, are captured by the simulation.^33^ The normalized node distances fit the limits of ${{\Delta }}y^{ + } = {{\Delta }}y \cdot u_{\tau } /\nu \ll 1$ ($< 4.4$ in core flow), ${{\Delta }}x^{ + } < 17.7$ and ${{\Delta }}z^{ + } < 5.9$, with y, x and z in wall-normal, streamwise and spanwise direction, respectively.^42^ We have ensured that the spatial and temporal discretization for the DNS calculations include the Kolmogorov scales. This led to a DNS mesh with 8 million elements. A second-order central differencing scheme was chosen for the spatial discretization and a second-order implicit scheme in time was used. The DNS was initialized with an already converged solution, in the sense of a statistically stationary state, and the time-averaging was done over a sufficiently long period of time so that all time-averaged turbulent statistics are 0.0. About 150k time steps with a fixed non-dimensional time step size ${{\Delta }}t \cdot u_{\tau } /h = 9.27 \cdot 10^{ - 4}$ are included within the results. All residuals were kept below $10^{ - 4}$ within inner coefficient loops. To validate the setup of our DNS, the mean velocity profile, Reynolds stresses and the TKE budget (only production and dissipation) were compared with DNS results from Moser *et al.*^33^,^42^ A snapshot of the instantaneous velocity magnitude and the mesh can be seen in Fig. 2.

#### Channel: Large Eddy Simulation

The mesh for the LES computations was adapted according to the recommendations from Fröhlich^14^ for wall-resolving LES (${{\Delta }}x^{ + } = \left( {{{\Delta }}x \cdot u_{\tau } } \right)/\nu < 50$, ${{\Delta }}z^{ + } < 15$ and ${{\Delta }}y^{ + } < 1$, including friction velocity $u_{\tau } = \left( {\tau_{w} /\rho } \right)^{0.5}$ and kinematic viscosity $\nu$). The LES mesh has a size of 1.2 million elements. To avoid any influence of an inappropriate temporal discretization, the same time step size as for the DNS was chosen for LES. The dynamic Smagorinsky model was chosen as sub-grid model and its constant was bounded between $C_{\text{S}} = \left( {0 \ldots 0.012} \right)$. The computation was initialized with the DNS results. The final simulation was running 170k time steps.

#### Channel: Reynolds-Averaged Navier–Stokes Simulation

In case for the RANS simulations, the turbulence was modeled utilizing the SST-$k$-$\omega$ (SST)^38^ and the $\omega$-Reynolds-stress (ORS)^56^ turbulence models. With these models, no turbulent movement is directly resolved, hence every turbulence influence on the mean flow field is modeled. The mesh from the DNS was used as a starting point and was scaled down in all directions with the only premise, that the first node in wall-normal direction lies within $y^{ + } < 1$ to avoid otherwise required wall functions. In means of grid elements, this led to a 20-times coarser mesh for SST and ORS computations with 400 thousand hexahedral elements. Mesh independence for the RANS setup is ensured and already demonstrated by means of an uncertainty quantification in previous studies^34^ using the Richardson extrapolation and approach proposed by Eça and Hoekstra.^7^ Both RANS computations were carried out until all residuals dropped below $10^{ - 8}$. The ORS model is a Reynolds stress model based on the omega-equation. This leads to a more accurate near wall treatment.

#### FDA Nozzle

**Figure 3:**
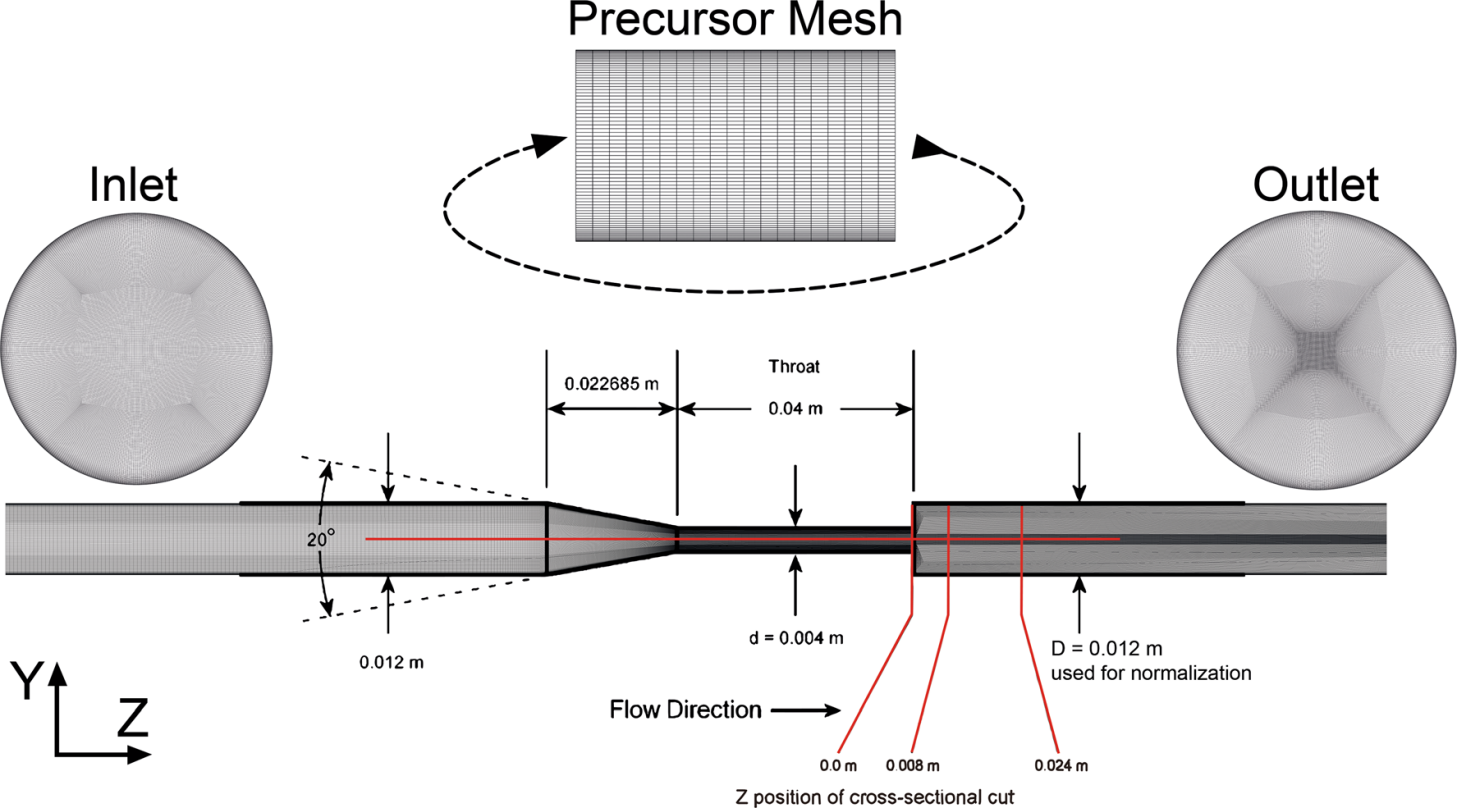
Used meshes for the FDA nozzle DNS and the precursor simulation for the inlet velocity profile. The Figure is adapted from Stewart *et al.*^26^ and the lines in red indicate locations where the simulations are validated and where the proposed equivalent stress is compared between the simulation methods. The large diameter *D* is used for normalization when comparing with MRI data.

The geometry, flow parameters and including fluid properties are taken from Stewart *et al.*^50^ We have chosen the case with the highest throat Reynolds number ${\text{Re}}_{t} = 6500$, built with smallest diameter and bulk velocity within the throat. Following the same scheme, an inlet Reynolds number of ${\text{Re}}_{i} = 2167$ is given. A velocity profile was implemented as an inlet boundary condition for all simulation methods. To create an inlet velocity profile which matches the literature data, a precursor simulation with a short section of the inlet pipe with a periodic inlet-outlet boundary conditions and the desired mass flow rate of $\dot{m} = 0.07149 \,{\text{kg/s}}$ was carried out. The meshes are shown in Fig. 3. The Spalart–Allmaras turbulence model^49^ for the precursor simulation was used in combination with an adjusted pressure update multiplier of 0.05, because it improved the iterative convergence of the solver. Here a coarser mesh with 115 thousand hexahedral elements and with $y^{ + } < 1$ was created. After 2453 iterations, the maximum residuals dropped below $2 \cdot 10^{ - 7}$ and the solver was manually stopped. The velocity profile calculated by the preliminary simulation was taken without additional artificial fluctuations as the inlet boundary condition for the following setups. We decided to use this approach since the inlet Reynolds number is relatively low and we assume a disturbance-free inlet flow. A zero static pressure was set at the outlet and all walls were set hydraulically smooth.

During post-processing, *x*-*y*-planes were set at selected *z*-positions. We transformed the Cartesian coordinates to cylindrical coordinates to make use of the axis symmetry and computed the average of variables of interest over constant radius. The results will therefore be shown as two-dimensional data of the time-averaged and three-dimensional flow field. Shown results along the axial length of the nozzle were obtained within post-processing by creating lines in the volume on which the respective variables were sampled along the length.

#### Nozzle: Direct Numerical Simulation

As for the channel, the normalized node distances were adjusted to fit the limits of ${{\Delta }}y^{ + } = {{\Delta }}y \cdot u_{\tau } /\nu \ll 1$ ($< 4.4$ in core flow), ${{\Delta }}x^{ + } < 17.7$ and ${{\Delta }}z^{ + } < 5.9$, with y, x and z in wall-normal, streamwise and spanwise direction, respectively. The DNS mesh has a size of 75 million elements. A second-order central differencing scheme was used for spatial discretization and an implicit second-order scheme in time was used. A maximum number of five inner loop iterations was allowed during solving, because it was expected that many time steps were needed for an appropriate time-averaging. However, maximum residuals lower than $10^{ - 5}$ are achieved in the area of interest. This resulted in an averaging time of about 200 thousand time steps. A time step of $1.4 \cdot 10^{ - 6} s$ was used.

#### Nozzle: Large Eddy Simulation

For LES the wall-adaptive local eddy-viscosity (WALE) model is used for the sub-grid scales as described in Reference 43. This model can be advantageous over the dynamic Smagorinsky model, since it does not require an explicit secondary filtering. Furthermore, it is capable to reproduce the laminar to turbulent transition. Obtained RANS results from this study were used to adapt the mesh to the recommendations of Fröhlich^14^ for wall-resolving LES. The final block-structured hexahedral mesh had 32 million elements. A bounded central differencing scheme was used for spatial discretization. This scheme blends from second-order to first-order accuracy in regions of steep gradients to prevent numerical oscillations. Other solver settings, properties and boundary conditions are identical with the DNS configuration.

#### Nozzle: Reynolds-Averaged Navier–Stokes Simulation

The flow through the nozzle was computed with RANS and SST model using a setup previously described in Reference 34. Since we use results for the nozzle that we have already generated for other publications, there are no results for the nozzle with Reynolds stress models. In the mentioned study, we have described the mesh independence of the setup by means of an uncertainty quantification using the Richardson extrapolation and approach proposed by Eça and Hoekstra.^7^

### Magnetic Resonance Imaging

Although the FDA nozzle geometry has been extensively studied in the literature, the experimental data base for validation is still limited. Currently, there is no experimental full-field flow data available in the previously published studies. For these reasons, we perform our own flow experiments to extend the data base for validation and increase the confidence in our numerical approach. Velocity-encoded phase-contrast MRI is chosen as measurement technique since it provides the mean velocity field in the entire flow domain at high accuracy and resolution.

The scientific basis for flow measurements with velocity-encoded MRI was established by Moran.^40^ Since then, MRI has been widely used in medicine, for example to measure the velocities of the blood flow in the human vascular system.^37^ Over the past decades there has been a considerable amount of work addressing MRI in the field of engineering and science. Comprehensive reviews on the possibilities and constraints of MRI are provided in Elkins and Alley^8^ and in Gladden and Sederman.^17^

In the most basic version, as applied in this study, velocity-encoded MRI provides the mean velocities inside fluid flows. MRI is non-intrusive and does not require optical access. The entire flow structure, including all boundary layers, can be captured within the discretization limits of the measurement resolution. Unlike many other flow measurement techniques, the MRI data contains no gaps within the measurement volume. For this reason, velocity-encoded MRI measurements are sometimes referred to as “CFD-grade experiments”. The numerical solution can be compared against the experimental data in the entire flow domain enabling a detailed validation of the numerical model.

In this study, velocity-encoded MRI measurements were conducted in the FDA nozzle to obtain an experimental reference for the axial mean velocity field. The measurements encompass the axial section from $z/D = - 0.3$ till $z/D = 2.3$ (see Fig. 2). The target Reynolds number is the same as in the DNS study (${\text{Re}} = 6500$). However, the physical dimensions were enlarged to achieve a higher measurement precision. The investigated FDA nozzle model had an inner diameter of $D = 0.0435 \,{\text{m}}$. The working fluid was pure water with a small concentration of MRI contrast agent (2g/L CuSO4) to allow fast imaging. The fluid temperature was controlled at the room temperature of $20\,^\circ {\text{C}}$, which yielded a kinematic viscosity of $1.00 \cdot 10^{ - 6} \,{\text{m}}^{ 2} / {\text{s}}$. With these values, a bulk velocity in the throat of $0.45 \,{\text{m/s}}$ is required to achieve ${\text{Re}} = 6500$. As a result, the target flow rate is $4.4 \,{\text{L/min}}$.

All MRI acquisitions were carried out on a 3 Tesla MRI system (Magnetom TRIO, Siemens Healthineers, Erlangen, Germany) with the magnetic field gradient system set to $38 \,{\text{mT/m}}$ and $170 \,{\text{T/m/s}}$. Two standard multi-channel receive-only surface coils provided by the vendor were used for acquiring the MR signal from the water. The flow rate was monitored with an ultrasound flow rate sensor (Deltawave C-F, Systec Controls, Puchheim, Germany),

The velocity field inside the FDA nozzle was acquired with a custom velocity-encoded gradient recalled echo sequence with a velocity sensitivity of $VENC = 1 \,{\text{m/s}}$. The measurement grid is Cartesian with an isotropic resolution of $\left( {0.5 \,{\text{mm}}} \right)^{3}$, which yields 87 data points across the diameter $D$. The spatial encoding axes were oriented such that a minimum amount of flow displacement occurred. i.e. the frequency-encoded axis was placed orthogonal to the flow direction (*z*), as this axis is most sensitive to flow displacement.^29^ The measurement was repeated eleven times and then averaged to increase the signal-to-noise ratio. Background phase errors were compensated by a reference acquisition without flow velocity (Flow Off). The Flow Off data was fitted onto Legendre polynomials of second degree to remove noise and then subtracted from the averaged velocity data. The main parameters of the MRI sequence are provided in Table 1.

**Table 1:** Main parameters of the MRI sequence.

Matrix size (*x*, *y*, *z*)	(256, 96, 256)
Spatial encoding type (x,y,z)	Frequency, phase, slice
Isotropic resolution in mm	0.5
Echo time in ms	4.1
Repetition time in ms	7.8
RF flip angle in °	35
Receiver bandwidth in Hz/pixel	331
VENC in m/s	1
Number of averages	11 with flow, 1 without flow
Total acquisition time in hours	2.6

The acquired data contains the velocity values and the image magnitude, which represents the strength of the magnetic resonance signal, i.e. high values represent regions with fluid and low values represent regions without fluid. The low-signal regions were removed by a manually determined threshold of the image magnitude. Next, the segmented Cartesian MRI data was transformed onto the cylindrical coordinates of the FDA nozzle and then averaged circumferentially.

The measurement uncertainty in the segmented Cartesian MRI data was estimated with the difference approach in Reference 4 The uncertainty value yields $0.016 \,{\text{m/s}}$, which is equivalent to $3.6 \%$ of the bulk velocity at the throat. The measurement uncertainty in the circumferentially averaged data depends on the number of samples at each radial position (*r*). From basic statistics, the measurement uncertainty in this data can be estimated as $0.016 \,{\text{m s}}^{ - 1} /\sqrt {2\pi r /0.5\, {\text{mm}}}$. For example, the measurement uncertainty at the maximum radial position yields only $0.2 \%$ of the bulk velocity at the throat.

The accuracy of the experiment was verified in following way. The volumetric flow rate was calculated from the segmented Cartesian MRI data by adding up the velocity values at each stream wise position (*z*). The mean of the calculated values is $1.1 \%$ higher than the target flow rate. The root mean square (RMS) deviation of the flow rate values of all streamwise positions is $1.2 \% .$ These deviations can be regarded negligible.

### Equivalent Scalar Stress

From a theoretical point of view, the instantaneous, acting mechanical stress is calculated by

1 $$\tau_{ij} = 2\mu S_{ij} = \mu \cdot \left( {\begin{array}{*{20}c} {2\frac{\partial u}{\partial x}} & {\left( {\frac{\partial u}{\partial y} + \frac{\partial v}{\partial x}} \right)} & {\left( {\frac{\partial u}{\partial z} + \frac{\partial w}{\partial x}} \right)} \\ {\left( {\frac{\partial v}{\partial x} + \frac{\partial u}{\partial y}} \right)} & {2\frac{\partial v}{\partial y}} & {\left( {\frac{\partial v}{\partial z} + \frac{\partial w}{\partial y}} \right)} \\ {\left( {\frac{\partial w}{\partial x} + \frac{\partial u}{\partial z}} \right)} & {\left( {\frac{\partial w}{\partial y} + \frac{\partial v}{\partial z}} \right)} & {2\frac{\partial w}{\partial z}} \\ \end{array} } \right)$$

with dynamic viscosity $\mu$ and rate-of-strain tensor $S_{ij}$. As shown in Eq. (1), fluid dynamic stress is described by a second-order tensor with nine components and velocity gradients in every direction. Respecting the symmetry of the tensor, the stress tensor has six independent components. It is obvious that a scalar equivalent stress, especially for further analysis regarding blood damage, would be advantageous over using Eq. (1). Most scalar stress formulations utilize the second invariant $II_{S}$ of the tensor^59^ which can also be expressed in terms of $S_{ij}$:

2 $$II_{S} = \frac{1}{2}\left( {tr\left( {\tau_{ij} } \right)^{2} - tr\left( {\tau_{ij}^{2} } \right)} \right) = - 2\mu^{2} S_{ij} S_{ij}$$

Assuming, that Eq. (2) appropriately represents multi-dimensional stress conditions described by the tensor of Eq. (1), a scalar equivalent stress magnitude can be derived:

3 $$\tau_{s} = \sqrt { - II} = \sqrt {2\mu^{2} S_{ij} S_{ij} }$$

As Hund *et al.*^26^ and Wu *et al.*^57^ stated out, all scalar equivalent stress formulations built upon the second invariant or using the norm of tensor $S_{ij}$, can be expressed with the physical quantity dissipation $\varepsilon$. Applying the definition of $\varepsilon = 2\nu S_{ij} S_{ij}$, with kinematic viscosity $\nu = \mu /\rho$, on Eq. (3), it follows:

4 $$\tau_{s} = \sqrt {2\mu^{2} S_{ij} S_{ij} } = \sqrt {\rho \mu \varepsilon }$$

With Eq. (4), the scalar equivalent stress, which can be formed with the dissipation and contains all information of the turbulence, can be implemented in a preferred numerical prediction model for blood damage. However, using dissipation requires highly detailed information of the turbulent flow.

For a full resolution of overall energy dissipation in the flow field, all length and time scales must be resolved. Which would result in a full near-wall resolution and temporal and spatial discretization fine enough to resolve down to the Kolmogorov scales, i.e. DNS. This is impracticable for blood damage prediction with CFD within VADs. In our opinion, the main reason for the inaccuracy of existing numerical blood damage models is the fact that RANS and LES simulations, that were applied in the investigations, do not include all scales of turbulence.

The relationship introduced before shown in Eq. (4) is the starting point for the derivation of the scalar equivalent stress, which should include every length scale of turbulent motion, without the need to resolve all scales of turbulent motion with a DNS.

For the analysis of turbulent flows, it is common practice to analyze the flow in a statistical framework. Therefore, the instantaneous variables of interest are decomposed into a mean and a fluctuating part. Following the Reynolds decomposition, the total instantaneous dissipation $\varepsilon_{\text{tot}}$ is decomposed into:

5 $$\begin{aligned} \varepsilon_{\text{tot}} &= \varepsilon_{\text{dir}} + \varepsilon_{\text{turb}} \hfill \\ \varepsilon_{\text{dir}} &= 2\nu \langle S_{ij} S_{ij}\rangle \hfill \\ \varepsilon_{\text{turb}} &= 2\nu S_{ij}^{'} S_{ij}^{'} \hfill \\ \end{aligned}$$

Here, triangle brackets constitute the time-averaged quantity and a dashed variable constitutes the respective fluctuation. In case of DNS results, the total dissipation $\varepsilon_{\text{tot}}$ contains the whole spectrum of turbulent movement, as well as the mean motion. In simulations with turbulence modeling, like RANS or LES, the spectrum is either not at all or only partially resolved and therefore Eq. (5) needs further treatment to cover all contributions to $\varepsilon_{\text{tot}}$. The share of resolved fluctuating velocity gradients is presented by $\varepsilon_{\text{turb,res}}$. The missing share of unresolved turbulent movement can be easily expressed in terms of the modeled turbulent dissipation $\varepsilon_{\text{turb,mod}}$. It is a direct solution variable in the used RANS (SST and ORS) models and can also be calculated from LES results by:

6 $$\varepsilon_{\text{turb,mod,LES}} = \nu_{{t,{\text{SGS}}}}|| S_{ij}^{2}||$$

with the eddy viscosity from the sub-grid-scale model $\nu_{{t,{\text{SGS}}}}$ and the norm of the rate-of-strain tensor.^45^ Combining this with Eq. (5), the total, time-averaged dissipation becomes:

7 $$\langle\varepsilon_{\text{tot}}\rangle = \varepsilon_{\text{dir}} + \langle\varepsilon_{\text{turb,res}}\rangle + \langle\varepsilon_{\text{turb,mod}}\rangle$$

Now, Eq. (7) can be inserted in Eq. (4) and the new definition of total equivalent scalar stress becomes:

8 $$\begin{aligned} \langle\tau_{\text{tot}}\rangle = \sqrt {\rho \mu \left( {\varepsilon_{\text{dir}} + \langle\varepsilon_{\text{turb,res}}\rangle + \langle\varepsilon_{\text{turb,mod}}\rangle } \right)} \hfill \\ \langle\tau_{\text{tot}}\rangle = \sqrt {2\mu^{2} \left( \langle{S_{ij} S_{ij}\rangle + \langle S_{ij}^{'} S_{ij}^{'}\rangle + \langle\varepsilon_{\text{turb,mod}}\rangle } \right)} \hfill \\ \end{aligned}$$

It is important, that the time-averaging is done before taking the root of the sum instead of averaging the whole equation for the stress. Otherwise, cross-terms, i.e. $S_{ij} S_{ij}^{'}$, are present in the stress computation. To analyze the influence of the resolution of turbulence in the flow simulations and on the stress computation, we separate the scalar equivalent stress Eq. (8) into following parts:

9 $$\begin{aligned} \tau_{\text{dir}} &= \sqrt {\rho \mu \varepsilon_{\text{dir}} } \hfill \\ \langle\tau_{\text{turb}}\rangle & = \sqrt {\rho \mu \left( {\langle\varepsilon_{\text{turb,res}}\rangle + \langle\varepsilon_{\text{turb,mod}}\rangle } \right)} \hfill \\ \langle\tau_{\text{turb,res}}\rangle & = \sqrt {\rho \mu \langle\varepsilon_{\text{turb,res}} \rangle} \hfill \\ \langle\tau_{\text{turb,mod}}\rangle & = \sqrt {\rho \mu \langle\varepsilon_{\text{turb,mod}} \rangle} \hfill \\ \end{aligned}$$

Covering the mechanical stress resulting from the mean flow field $\tau_{\text{dir}}$, the mechanical stress emerging from time-averaged resolved turbulent scales $\langle\tau_{\text{turb,res}}\rangle$ and from modeled scales $\langle\tau_{\text{turb,mod}}\rangle$. The stresses were calculated with Eqs. (8) and (9) in the flow fields of the simulations with turbulence modeling and compared with the baseline stresses from DNS to verify, whether the new scalar stress formulation, including the non-resolved term, covers the influence of modeled turbulent movement correctly. In order to compare the calculation of stresses not only locally between the simulation and calculation methods, global values should also be included in the evaluation.

In addition to a local comparison of stress calculation between the simulation methods, a simple volume integral $I$ over the scalar equivalent stresses shall be compared.

10 $$\begin{aligned} I\left( \langle{\tau_{\text{tot}} \rangle} \right) &= \mathop \int \limits_{V} \langle\tau_{\text{tot}\rangle} dV = \mathop \int \limits_{V} \sqrt {2\mu^{2} \left( \langle{S_{ij} S_{ij}\rangle + \langle S_{ij}^{'} S_{ij}^{'}\rangle + \langle\varepsilon_{\text{turb,mod}}\rangle } \right)} dV \hfill \\ I\left( {\tau_{\text{dir}} } \right) &= \mathop \int \limits_{V} \tau_{\text{dir}} dV = \mathop \int \limits_{V} \sqrt {\rho \mu \varepsilon_{\text{dir}} } dV \hfill \\ I\left( {\langle\tau_{\text{turb}}\rangle } \right) &= \mathop \int \limits_{V} \langle\tau_{\text{turb}}\rangle dV = \mathop \int \limits_{V} \sqrt {\rho \mu \left( {\langle\varepsilon_{\text{turb,res}}\rangle + \langle\varepsilon_{\text{turb,mod}}\rangle } \right)} dV \hfill \\ \end{aligned}$$

With this the global influence of the modeled field shall be analyzed. Since the sizes of the computational volumes for the FDA nozzle differ for the individual simulation methods in means of length, only the volume between $Z = \left( { - 0.088 \ldots 0.08} \right) \,{\text{m}}$ is integrated. In addition, the integral of the stresses is often used indirectly to estimate blood damage as a first evaluation step This comparison is thus intended to provide insight into how the stress calculation might affect global blood damage estimates.

As a final method, the influence of turbulence consideration in the stresses on the calculation of the modified index of hemolysis $MIH$ shall be demonstrated. However, it should be said that this method is not intended to calculate the potential blood damage from the flows, but only to demonstrate the influence on the $MIH$ calculation. This is to avoid the impression that our method improves blood damage prediction per se. For this goal, fundamentally improved prediction models are needed. The model is based on a scalar transport equation, where the production and transport of plasma-free hemoglobin $H$, i.e. due to hemolysis, is modeled.^59^ We have intentionally chosen such a simple model because it is especially these models that are used in the first design and evaluation steps of a VAD.

11 $$\begin{aligned} \frac{{\partial H_{L} }}{\partial t} + \frac{{\partial \left( {u_{i} H_{L} } \right)}}{{\partial x_{i} }} = S \hfill \\ S = C^{{\frac{1}{\beta }}} \tau_{s}^{{\frac{\alpha }{\beta }}} \hfill \\ \end{aligned}$$

Equation (11) includes the linearized plasma-free hemoglobin $H_{L} = H^{1/\beta }$ and production term $S$, which depends on the experimentally determined constants $C, \alpha$ and $\beta$ and a scalar equivalent stress $\tau_{s}$. We have chosen the constants from the experiments of Zhang *et al.*^61^ with $1.228 \cdot 10^{ - 7}$, $1.9918$ and $0.6606$ for $C$, $\alpha$ and $\beta$, respectively. The solution of a transport equation for hemolysis prediction was first introduced by Garon and Farinas.^15^ Solving an additional transport equation requires more computational resources and this can be a sensitive factor, especially in the case of DNS. Therefore, we use common simplifications for Eq. (10), which seem to be appropriate for the given flow cases. The simplifications are the assumption that the flow variables are statistically constant over time and that the information of evolved plasma-free hemoglobin is only needed at the outlet of the domain.^15^ Following the assumptions, Eq. (10) can be rewritten as:

12 $$\mathop \int \limits_{V} \frac{{\partial \left( {u_{i} H_{L} } \right)}}{{\partial x_{i} }}{\text{d}}V = \mathop \int \limits_{V} S{\text{d}}V$$

Now the integral over the domain volume $V$ on the left side is transformed into a surface integral over the whole surface $A$ of the volume $V$:

13 $$\mathop \int \limits_{A} u_{i} \cdot n_{i} H_{L} {\text{d}}A = \mathop \int \limits_{V} S{\text{d}}V$$

With surface normal vector $n_{i}$. The integral over the walls become zero due to the scalar product $u_{i} \cdot n_{i}$. No plasma-free hemoglobin enters the computational domain at the inlet and the integral at the inlet also becomes zero. The surface integral on the left-hand-side of Eq. (13) only reduces to the integral across the outlet area of the computational domain. The global time-average hemolysis indicator $\bar{H}$ can be computed by:

14 $$\begin{aligned} \bar{H}_{L} = \frac{1}{Q}\mathop \int \limits_{V} S{\text{d}}V = \frac{1}{Q}\mathop \int \limits_{V} C^{{\frac{1}{\beta }}} \tau_{s}^{{\frac{\alpha }{\beta }}} {\text{d}}V \hfill \\ \bar{H} = \bar{H}_{L}^{\beta } \hfill \\ \end{aligned}$$

With $Q \left[ {m^{3} /s} \right]$ as the volume flux of the blood flow through the domain. For better readability and comparability of the results the modified index of hemolysis $MIH = 10^{6} \cdot \bar{H}$ was calculated according to Reference 15.

To investigate the influence of turbulence, i.e. the resolved and the modeled components, on the $MIH$ calculation, the different stresses from Eq. (9) were used to calculate the hemolysis indicator according to Eq. (14). This way it can be investigated how the prediction of blood damage changes for the results of simulations with turbulence modeling methods (RANS and LES) when the modeled shares of the dissipation are taken into account. The final equations for comparing the blood damage prediction can be summed up as follows:

15 $$\begin{aligned} MIH\left( {\tau_{\text{dir}} } \right) &= 10^{6} \cdot \left( {\frac{1}{Q}\mathop \int \limits_{V} C^{{\frac{1}{\beta }}} \tau_{\text{dir}}^{{\frac{\alpha }{\beta }}} dV} \right)^{\beta } \\ & = 10^{6} \cdot \left( {\frac{1}{Q}\mathop \int \limits_{V} C^{{\frac{1}{\beta }}} \left( {\sqrt {\rho \mu \varepsilon_{\text{dir}} } } \right)^{{\frac{\alpha }{\beta }}} dV} \right)^{\beta }\\ MIH\left( {\langle\tau_{\text{turb}}\rangle } \right) &= 10^{6} \cdot \left( {\frac{1}{Q}\mathop \int \limits_{V} C^{{\frac{1}{\beta }}} \langle\tau_{\text{turb}}\rangle^{{\frac{\alpha }{\beta }}} dV} \right)^{\beta } \\ &= 10^{6} \cdot \left( {\frac{1}{Q}\mathop \int \limits_{V} C^{{\frac{1}{\beta }}} \left( {\sqrt {\rho \mu \left( {\langle\varepsilon_{\text{turb,res}}\rangle + \langle\varepsilon_{\text{turb,mod}}\rangle } \right)} } \right)^{{\frac{\alpha }{\beta }}} dV} \right)^{\beta } \hfill \\ MIH\left( {\langle\tau_{\text{tot}}\rangle } \right) &= 10^{6} \cdot \left( {\frac{1}{Q}\mathop \int \limits_{V} C^{{\frac{1}{\beta }}} \langle\tau_{\text{tot}}\rangle^{{\frac{\alpha }{\beta }}} dV} \right)^{\beta }\\ &= 10^{6} \cdot \left( {\frac{1}{Q}\mathop \int \limits_{V} C^{{\frac{1}{\beta }}} \left( {\sqrt {\rho \mu \left( {\varepsilon_{\text{dir}} + \langle\varepsilon_{\text{turb}}\rangle + \langle\varepsilon_{\text{turb,mod}}\rangle } \right)} } \right)^{{\frac{\alpha }{\beta }}} dV} \right)^{\beta }\end{aligned}$$

Again, only the volume between $Z = \left( { - 0.088 \ldots 0.08} \right)\,{\text{m}}$ is integrated for this calculation.

## Results

### Validation

To validate the DNS, we compared the mean static pressure along the centerline (nozzle), velocity profiles (channel and nozzle), shear stress profiles (nozzle), Reynolds stress components (channel), as well as dissipation and production from the TKE budget (channel) with data from the literature: Moser *et al.*^42^ (channel) and Stewart *et al.*^50^ (nozzle) and with experimental MRI data (nozzle). All shown CFD results are time-averaged quantities.

#### Channel Flow

**Figure 4:**
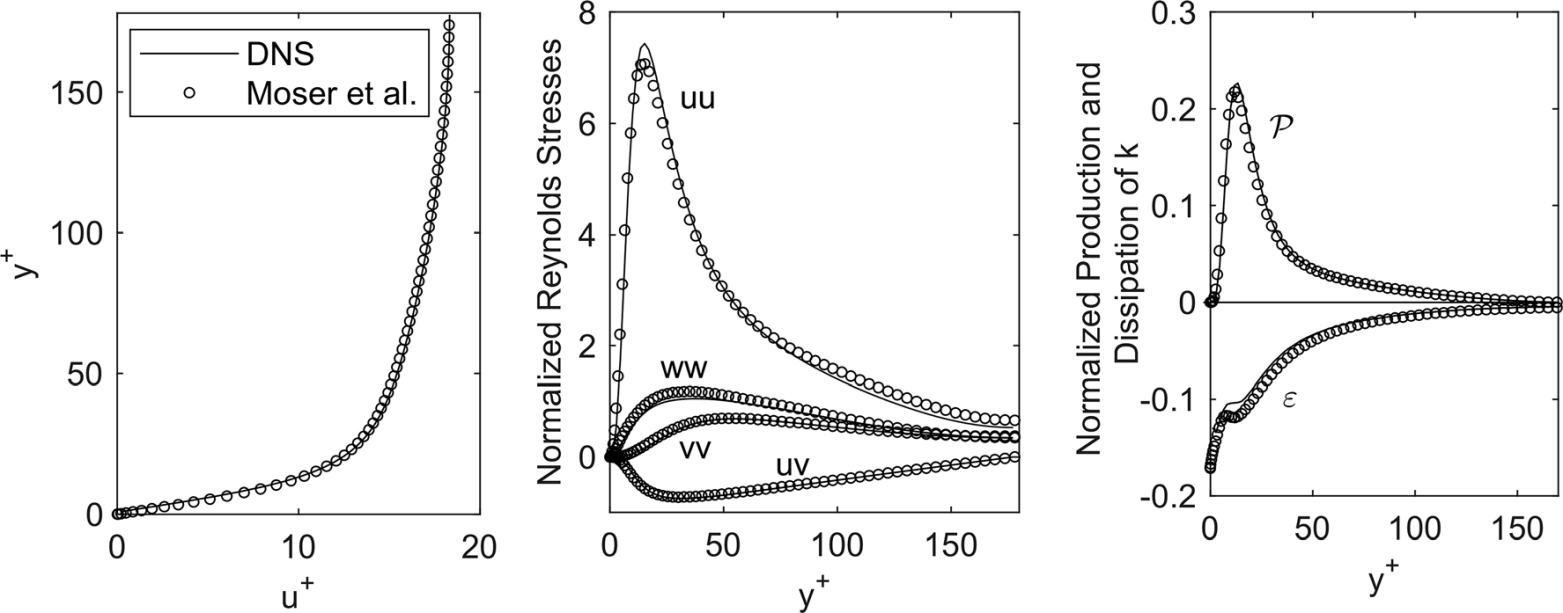
From left to right: Comparison of mean velocity profile, Reynolds stress components, as well as production ${\mathcal{P}}$ and dissipation $\varvec{\varepsilon}$ from turbulence kinetic energy budget between literature data (circles)^33^ and DNS results (solid lines).

The results show that the Courant number ranges between $0.01$ and $0.8$ ($0.005$ and $0.3$ for LES). All results are made dimensionless, with $u_{\tau }$ and $\nu$ as shown in Fig. 4. They show a very good agreement with the data from Moser *et al.* From the comparison of Reynolds stresses and TKE budget, one can conclude, that the mesh size is adequate and that the turbulent data from the DNS can serve as a baseline for the comparison with the results from the simulations with turbulence modeling.

#### FDA Nozzle

In general, the ratio between grid size and Kolmogorov length scale ranges between $V^{1/3} /\left( {\nu^{3} /\varepsilon_{turb} } \right)^{1/4} = \left( {0.008 \ldots 0.9} \right)$, with $V$ as the volume of the hexahedral grid cell. The maximum value is found in the break up zone of the jet. The Courant number ranges between $4 \cdot 10^{ - 7}$ and $0.9$ ($4 \cdot 10^{ - 7}$ and $0.3$ for LES). In addition to the validation with experimental data from the literature we validate the DNS with experimental data from MRI measurements. A special focus is put on the velocity profiles in the area right downstream of the sudden expansion. Since the physical dimensions were different in the experiment and simulation, the data was compared in non-dimensional form. The geometry and velocity were normalized with $D$ and the bulk velocity in the throat $U_{\text{throat}}$.

The inlet boundary conditions were validated using the static pressure and the wall shear stress along the axial length, and the velocity profile at the inlet of the experimental literature data from Stewart *et al.* Additionally the profiles of the shear stress at $Z = \left( {0.008;0.024} \right) \,{\text{m}}$ were compared.

**Figure 5:**
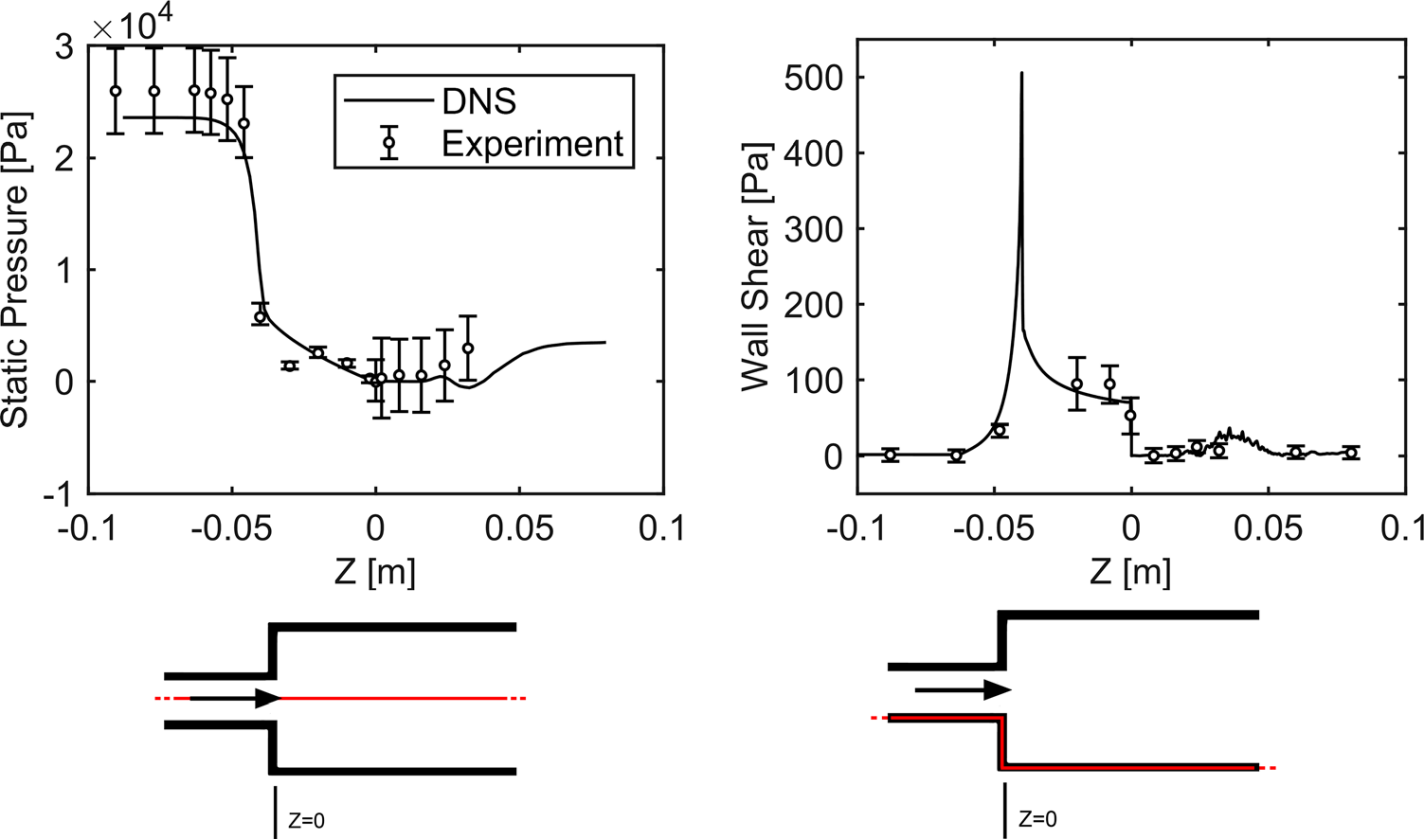
Comparison between literature data and its uncertainties from Stewart *et al.* (circles)^33^ and DNS results (solid lines) for static pressure along centerline (left) and wall shear stress along *z*-coordinate (right). The red lines in the bottom sketches only indicate the origin of the data.

**Figure 6:**
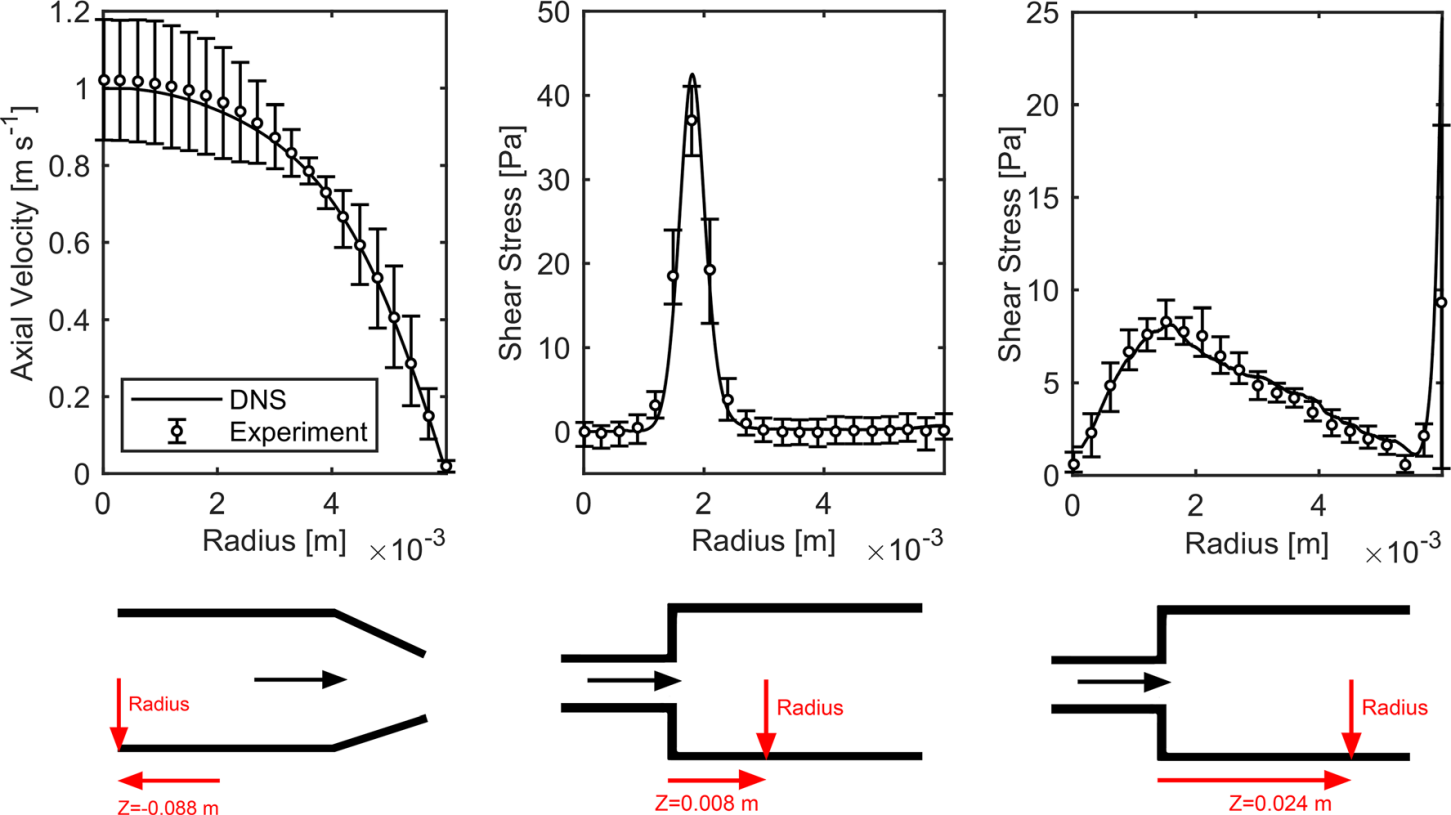
Comparison of literature data and its uncertainties from Stewart *et al.* (circles) and DNS results (solid lines) in means of inlet velocity profile (left), shear stress profile at $Z = 0.008 \,{\text{m}}$ (middle) and shear stress profile at $Z = 0.024 \,{\text{m}}$ (right).

The static pressure along the centerline and the wall shear stress along the *z*-coordinate are shown in Fig. 5. With few exceptions, the DNS data fall within the uncertainties of the literature data. The profiles of inlet velocity and shear stresses at the positions $Z = \left( {0.008;0.024} \right) \,{\text{m}}$ are shown in Fig. 6. The inlet velocity profile matches almost perfectly with the experimental data. The profiles of shear stress at the two cuts also agree very well with the experimental results from the literature. Where exceedingly high stresses are present, e.g. on the wall in the downstream cut or in the peak of the shear layer further upstream, the DNS results indicate higher values.

**Figure 7:**
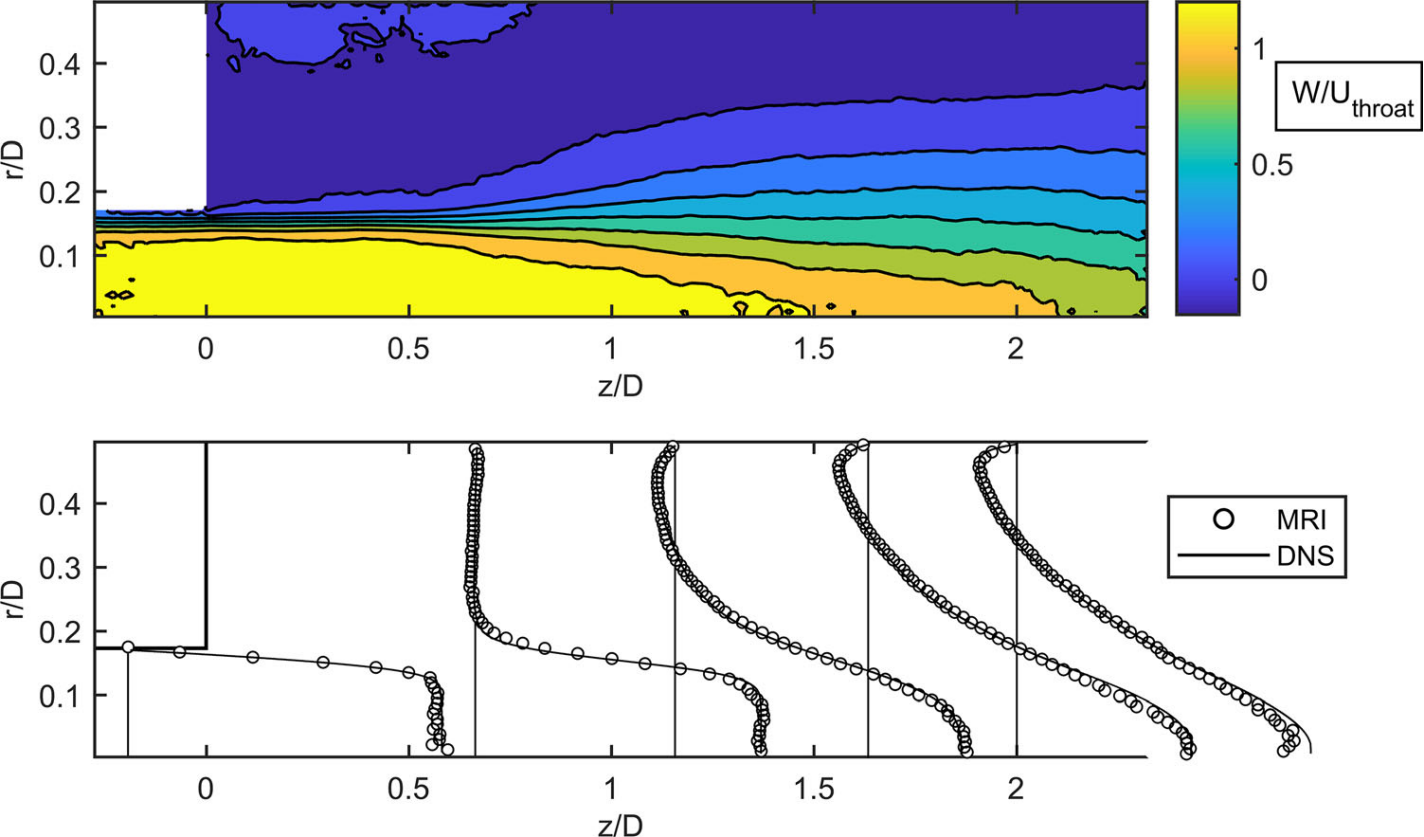
Time-averaged axial velocity after the sudden expansion for the FDA case measured with MRI (top). Comparison of MRI and DNS velocity at selected cuts $z/D = \left( { - 0.1;0.7;1.1;1.6;2} \right)$ (bottom).

The measured MRI data and the comparison with velocity profiles at selected cuts are shown in Fig. 7. The circumferentially averaged data of the measured three-dimensional flow field downstream of the sudden expansion is shown at the top. A jet can be seen, which expands to the end of the measurement window. In the upper, left area of the nozzle downstream the expansion, is a large area with a normalized velocity of near zero. The comparison between measured and calculated velocity profiles shows an exceptionally good agreement between MRI and DNS. A mean absolute deviation of $\sum (W_{\text{DNS}}^{*} - W_{\text{MRI}}^{*} )/N = 0.004$, with normalized axial velocities $W^{*} = W/W_{\text{throat}}$ and number of data points $N$, can be calculated. We conclude that the DNS of the flow in the nozzle can be used as a baseline for the shear stress and blood damage prediction of the simulations with turbulence modeling.

### Shear Stress Computation

#### Channel Flow

**Figure 8:**
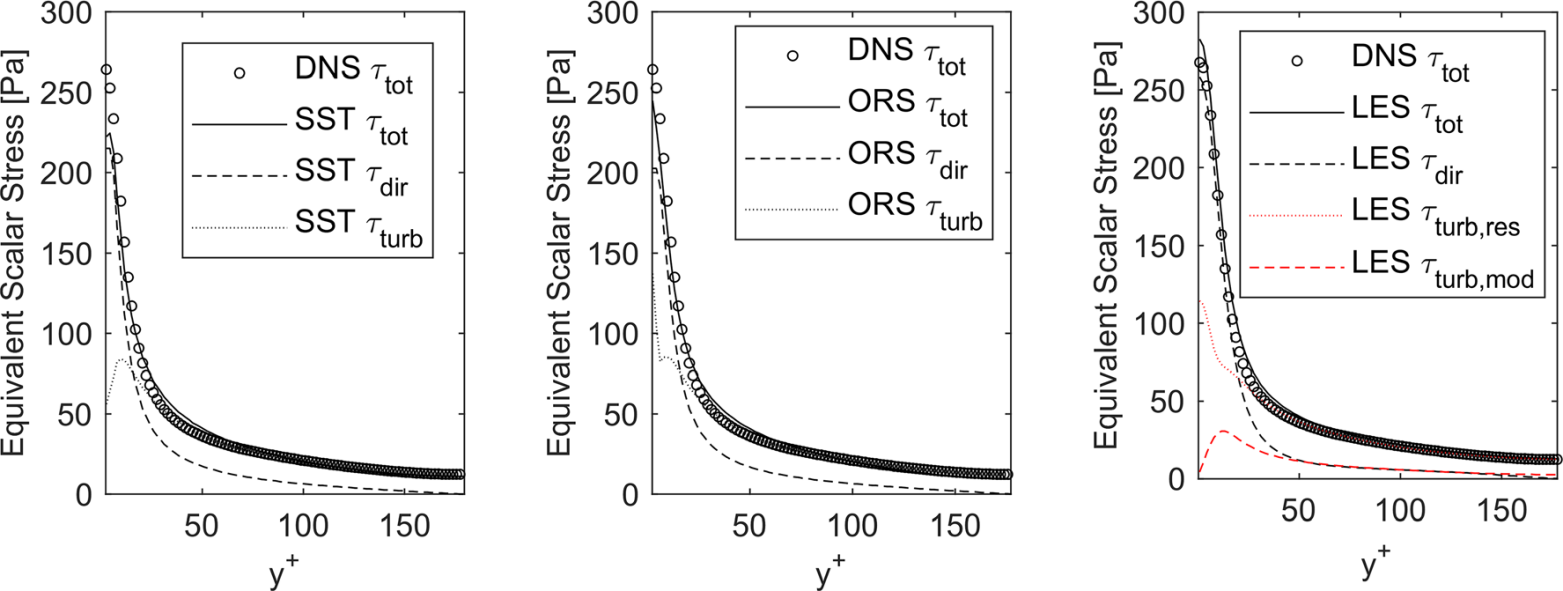
Comparison of calculated total scalar stress ${{\tau }}_{\text{tot}}$ and respective shares (mean stress from direct dissipation ${{\tau }}_{\text{dir}}$, turbulent stress including resolved ${{\tau }}_{{{\text{turb,res}}}}$ and modeled ${{\tau }}_{\text{turb,mod}}$ part) for the turbulent channel flow. DNS versus SST (left), DNS versus ORS (middle) and DNS versus LES (right).

With validated DNS data, we calculated the shear stress field using Eq. (8) (but without $\tau_{\text{turb,mod}}$ obviously) to obtain an equivalent scalar stress $\tau_{\text{tot}}$. This mechanical scalar stress contains all spatial and temporal scales of the flow. Following Eq. (8) and Eq. (9), we calculated the same field for the RANS SST and ORS results. No turbulent fluctuation is resolved in these simulations and every influence of turbulence in the equivalent stress is coming from the respective turbulence model $\tau_{\text{turb,mod}}$. In the same manner, the total stress for the LES was calculated, except for the turbulent share of the scalar stress, which has two components now: the modeled $\tau_{\text{turb,mod}}$ and the resolved part $\tau_{\text{turb,res}}$. The comparison between the results is shown in Fig. 8.

As the most important result, the total stress, as the sum of direct and turbulent mechanical stresses, is in a good agreement between all simulations with turbulence modeling and DNS. Now, the different contributions to the total stress for all computations can be compared. When only direct stresses $\tau_{\text{dir}}$, coming from direct dissipation, are considered, the scalar equivalent stress would be substantially underpredicted and especially in the core region of the flow and for all computations. If the turbulent share $\tau_{\text{turb}}$ is added to the direct stress, the agreement becomes substantially better when compared to the baseline stress from DNS. However, for the SST results, a significant under-prediction of total stress is noticeable in the vicinity of the wall. ORS and LES are performing better. Although $\tau_{\text{dir}}$ is comparable between SST and ORS, the modeled turbulent stress $\tau_{\text{turb,mod}}$ shows higher values at the wall for the ORS model and thus, the total stress compares much better at the wall with the DNS. In general, the better accuracy of Reynolds stress models is apparent which is grounded in more degrees of freedom by solving additional transport equation for every independent Reynolds stress component. The turbulent share for LES is divided into resolved $\tau_{\text{turb,res}}$ and modeled part $\tau_{\text{turb,mod}}$. The resolved stress is comparable with the modeled turbulent stress from the ORS results. A slight over-prediction of total stress at the wall is visible for LES, which is a common known issue of using LES.^5^

#### FDA Nozzle

**Figure 9:**
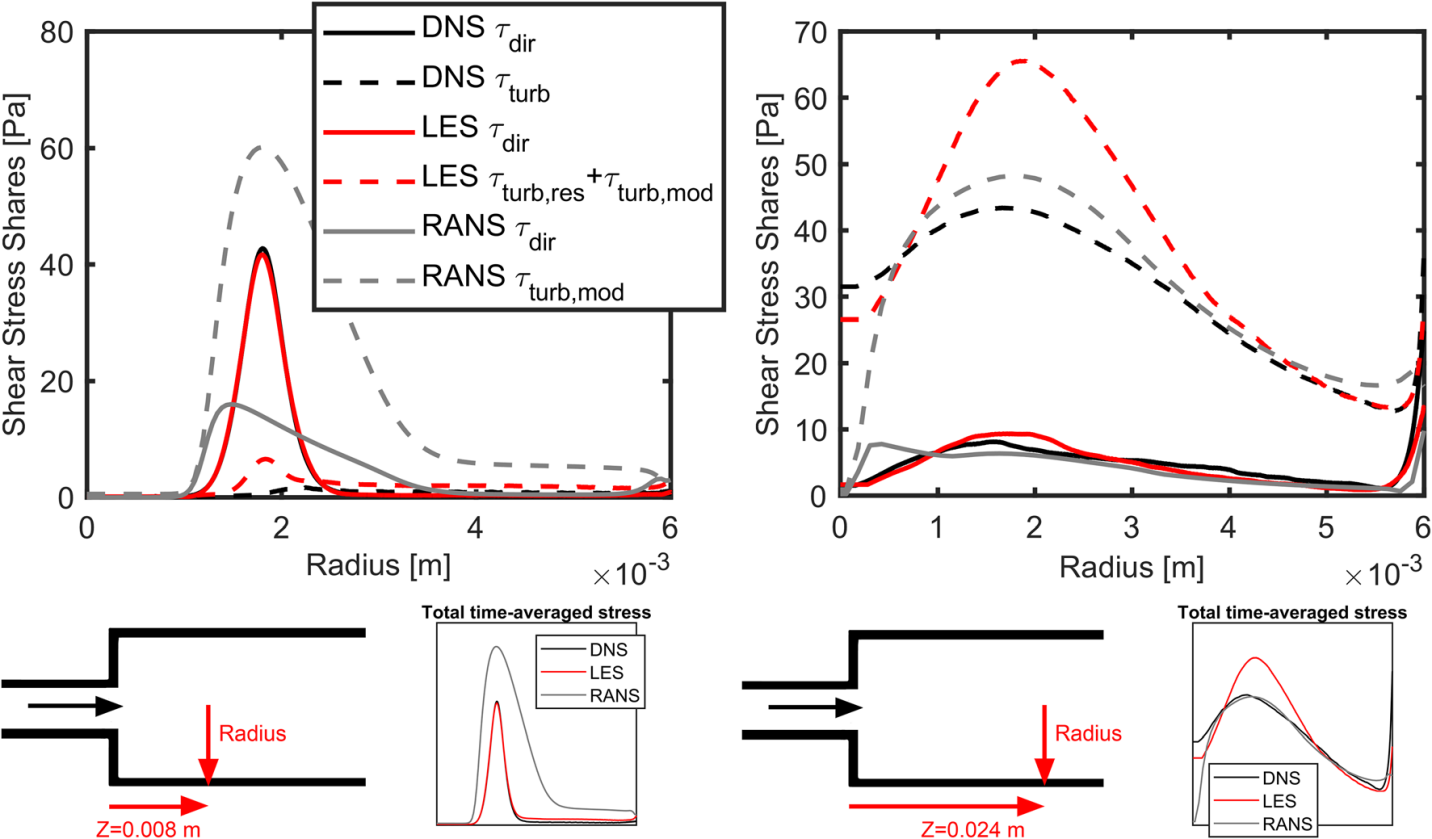
Comparison of calculated scalar stress shares and total stress between DNS, LES and RANS results for the FDA nozzle case.

We compare the scalar equivalent stresses calculated with Eq. (4) for DNS and with Eq. (8) and Eq. (9) for RANS and LES results at two positions $Z = 0.008 \,{\text{m}}$ and $Z = 0.024$ m. The results can be found in Fig. 9. In general, it is clearly visible that the contribution of turbulence to the total stress is much higher downstream. In contrast, upstream there is a dominant shear layer between the jet and surrounding fluid, which does not seem to be broken up by instabilities, yet. In the left figure in grey one can see that the fields of mechanical stress between RANS and the other methods differ strikingly. In contrast, the direct stresses between LES and DNS match very well in both figures. Only the turbulent part to the total stress is slightly overestimated by LES, although the absolute amount is still small. Downstream, where the shear layer is broken up and turbulent mixing takes place, the stress fields between all methods are more comparable. It is worth mentioning how close the turbulent stresses between DNS and RANS match. Only in the core of the flow they decrease rapidly for RANS. The turbulent part of the stresses in the center is better reproduced by LES, whereas they are again overestimated with increasing radius.

### Overall Stress and MIH Calculation

The results of the computed volume integrals $I$ using only turbulent stresses, direct stresses and total stresses can be found in Table 2 for the channel flow and in Table 3 for the nozzle. It becomes clear that $I$, if it is formed only from the mean field of RANS simulations, is far below the values for DNS. In RANS calculations, $\tau_{\text{dir}}$ includes the complete, resolved field. It is also visible that the integral over $\tau_{\text{dir}}$ is smaller for the RANS models than for LES and DNS. If the modeled field is taken into account, the $I$ values of under-resolved methods are all close to DNS. In the case of the channel, the values are about twice as high as when only the resolved flow is considered.

**Table 2:** Calculated $I$ solely from the mean stresses, only from the turbulent stresses and the total field with Eq. (10) for the channel flow using different turbulence modeling approaches.

Channel flow	$I\left( {\tau_{\text{dir}} } \right) \cdot 10^{ - 5}$ [J]	$I\left( {\langle\tau_{\text{turb}}\rangle } \right) \cdot 10^{ - 5}$ [J]	$I\left( {\langle\tau_{\text{tot}}\rangle } \right) \cdot 10^{ - 5}$ [J]
DNS	1.46	1.67	2.01
LES	1.43	1.69	2.19
ORS	0.85	1.64	1.92
SST	0.86	1.62	1.93

Please note that the sum of the individual stress components for $\langle\tau_{\text{tot}}\rangle$ is formed under the square root and therefore $I\left( {\langle\tau_{\text{tot}}\rangle } \right) = I\left( {\sqrt {\rho \mu \left( {\tau_{\text{dir}} + \langle\tau_{\text{turb}}\rangle } \right)} } \right) \ne I\left( {\tau_{\text{dir}} } \right) + I\left( {\langle\tau_{\text{turb}}\rangle } \right)$

**Table 3:** Calculated $I$ solely from the mean stresses, only from the turbulent stresses and the total field with Eq. (10) for the channel flow using different turbulence modeling approaches.

FDA nozzle	$I\left( {\tau_{\text{dir}} } \right) \cdot 10^{ - 5}$ *[J]*	$I\left( {\langle\tau_{\text{turb}}\rangle } \right) \cdot 10^{ - 5}$ *[J]*	$I\left( {\langle\tau_{\text{tot}}\rangle } \right) \cdot 10^{ - 5}$ *[J]*
DNS	3.90	9.67	10.4
LES	3.40	9.49	10.1
SST	3.03	9.23	9.71

Please note that the sum of the individual stress components for $\langle\tau_{\text{tot}}\rangle$ is formed under the square root and therefore $I\left( {\langle\tau_{\text{tot}}\rangle } \right) = I\left( {\sqrt {\rho \mu \left( {\tau_{\text{dir}} + \langle\tau_{\text{turb}}\rangle } \right)} } \right) \ne I\left( {\tau_{\text{dir}} } \right) + I\left( {\langle\tau_{\text{turb}}\rangle } \right)$

For the nozzle, the acting stresses are generally higher than for the channel. While $I$ from the direct stress is usually lower for all simulation methods, the influence of turbulence to the total stress field is higher for all nozzle simulations than for the channel. In addition, the overprediction of $\tau_{\text{turb}}$ in the channel by LES becomes again visible here. The integral $I$ built with the total stress is always lower when computed from turbulence modeling methods in comparison to DNS (as it can be seen in Tables 2 and 3).

**Figure 10:**
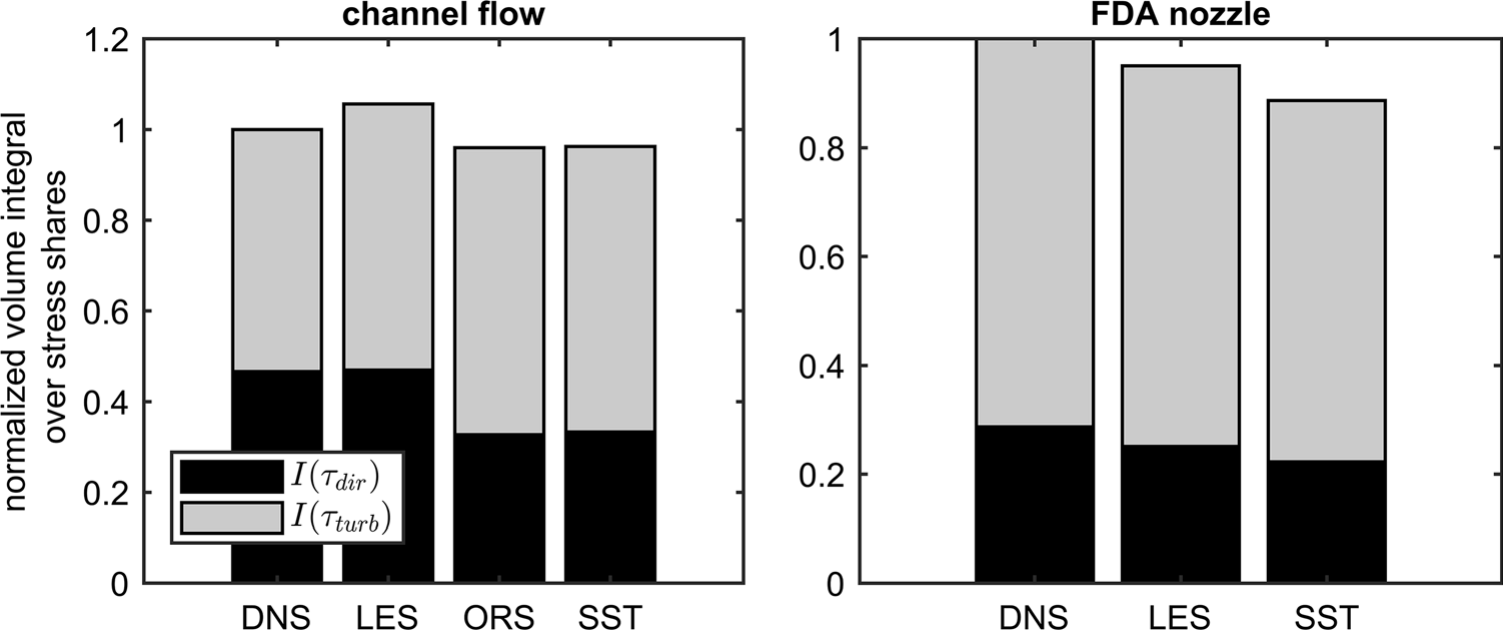
Comparison of calculated sum of $I\left( {\tau_{\text{dir}} } \right) + I\left( {\langle\tau_{\text{turb}}\rangle } \right)$ and its shares between the used turbulence modeling approaches and DNS for the channel flow and nozzle. The normalized $I\left( {\tau_{i} } \right)_{{j,{\text{norm}}}}$ of $j$-th simulation method with $i$-th stress is calculated by $I\left( {\tau_{i} } \right)_{{j,{\text{norm}}}} = I\left( {\tau_{i} } \right)_{j} \cdot I\left( {\tau_{\text{tot}} } \right)_{j} /\left( {I\left( {\tau_{\text{tot}} } \right)_{\text{DNS}} \cdot \left( {I\left( {\tau_{\text{dir}} } \right)_{j} + I\left( {\tau_{\text{turb}} } \right)_{j} } \right)} \right)$ to visualize the shares as a pseudo-sum of mean and turbulent share.

The results are summarized in Fig. 10 for better comparability. All values were normalized for comparability with the respective DNS.

**Figure 11:**
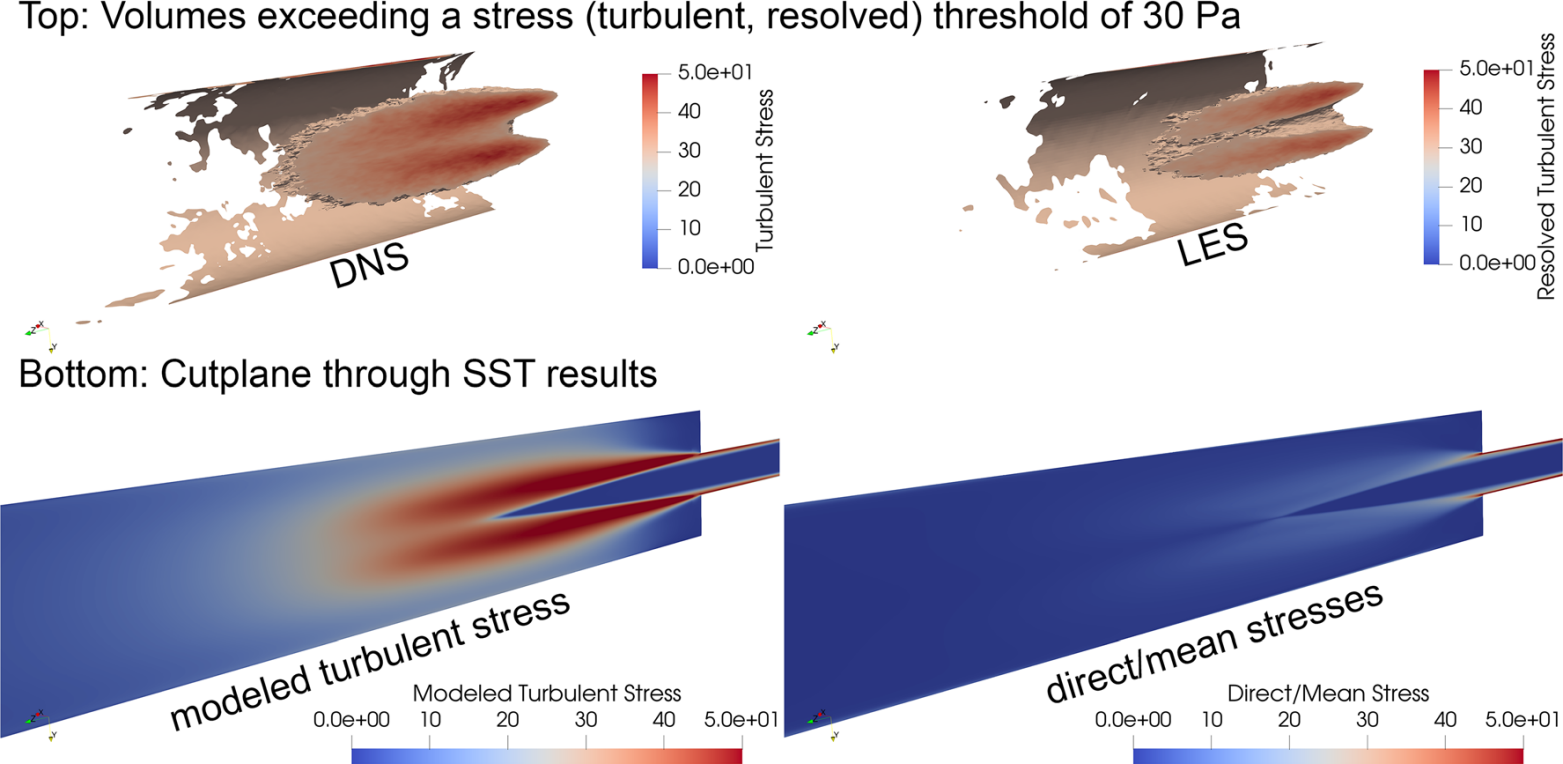
Local comparison of stress calculation in the nozzle between used equivalent stress and simulation method. Top: Volumes within DNS and LES results where a threshold of 30 Pa of time-averaged resolved turbulent stress was exceeded (volumes clipped by midplane). Bottom: cut planes through SST results representing modeled turbulent and mean stresses between 0 and 50 Pa.

To illustrate the local differences in the stress calculation, Fig. 11 shows the calculation of the different shares in the nozzle and between the different simulation methods. The two sub-pictures above show volumes in which turbulent, resolved stresses above 30 Pa occur on time average. For better visibility, the volumes have been clipped through the midplane. In addition, the scale shows (resolved) turbulent stresses between 0 and 50 Pa. First of all, it is visible that most turbulent stresses occur in the mixing layer between the jet and the surrounding region, the recirculation zone. By applying only the resolved stresses, which in the case of the DNS corresponds to the complete stress field, the better resolving capability of the DNS compared to the LES is again shown here, since an overall larger volume above 30 Pa is displayed.

The lower two images in are intended to illustrate once again the difference between the modeled and the mean stresses in the case of the RANS simulation. The viewpoint is the same in all four images. The direct/mean stresses of the RANS results correspond to the total resolved field. It is visible that only in the boundary layers at the wall stresses above 50 Pa are reached. If the modeled stresses on the left are considered, there are now clear quantitative similarities to the achieved stress values of the much more elaborate LES and DNS results.

The results of the computed $MIH$ values using only turbulent stresses, direct stresses and total stresses can be found in Table 4 for the channel flow and in Table 5 for the nozzle. It becomes clear that the $MIH$, if it is formed only from the mean field of RANS simulations, is far below the values for DNS. In RANS calculations, $\tau_{\text{dir}}$ includes the complete, resolved field. It is also visible that the blood damage from $\tau_{\text{dir}}$ is smaller for the RANS models than for LES and DNS. If the modeled field is taken into account, the $MIH$ values of under-resolved methods are all close to DNS. In the case of the channel, the values are about twice as high as when only the resolved flow is considered.

**Table 4:** Calculated $MIH$ solely from the mean stresses, only from the turbulent stresses and the total field with Eq. (14) for the channel flow using different turbulence modelling approaches.

Channel flow	$MIH\left( {\tau_{\text{dir}} } \right)$	$MIH\left( {\langle\tau_{\text{turb}}\rangle } \right)$	$MIH\left( {\langle\tau_{\text{tot}}\rangle } \right)$
DNS	20.16	6.33	25.15
LES	20.27	4.89	24.25
ORS	11.77	10.43	21.27
SST	12.56	12.56	19.79

Please note that the sum of the individual stress components for $\langle\tau_{\text{tot}}\rangle$ is formed under the square root and therefore $MIH\left( {\langle\tau_{\text{tot}}\rangle } \right) = MIH\left( {\sqrt {\rho \mu \left( {\tau_{\text{dir}} + \langle\tau_{\text{turb}}\rangle } \right)} } \right) \ne MIH\left( {\tau_{\text{dir}} } \right) + MIH\left( {\langle\tau_{\text{turb}}\rangle } \right)$

**Table 5:** Calculated $MIH$ solely from the mean stresses, only from the turbulent stresses and the total field with Eq. (14) for the FDA nozzle using different turbulence modelling approaches.

FDA nozzle	$MIH\left( {\tau_{\text{dir}} } \right)$	$MIH\left( {\langle\tau_{\text{turb}}\rangle } \right)$	$MIH\left( {\langle\tau_{\text{tot}}\rangle } \right)$
DNS	14.86	18.06	32.34
LES	14.69	18.14	31.52
SST	9.64	17.75	30.66

Please note that the sum of the individual stress components for $\langle\tau_{\text{tot}}\rangle$ is formed under the square root of Eq. (14) and therefore $MIH\left( {\langle\tau_{\text{tot}}\rangle } \right) = MIH\left( {\sqrt {\rho \mu \left( {\tau_{\text{dir}} + \langle\tau_{\text{turb}}\rangle } \right)} } \right) \ne MIH\left( {\tau_{\text{dir}} } \right) + MIH\left( {\langle\tau_{\text{turb}}\rangle } \right)$

For the nozzle, the predicted blood damage calculated from the entire field is generally higher than for the channel. While the $MIH$ from the direct stress is usually lower for all simulation methods, the blood damage that would result from the turbulent field is generally higher for all cases than for the channel. In addition, the $MIH$ built with the total stress is always lower when computed from turbulence modeling methods in comparison to DNS.

**Figure 12:**
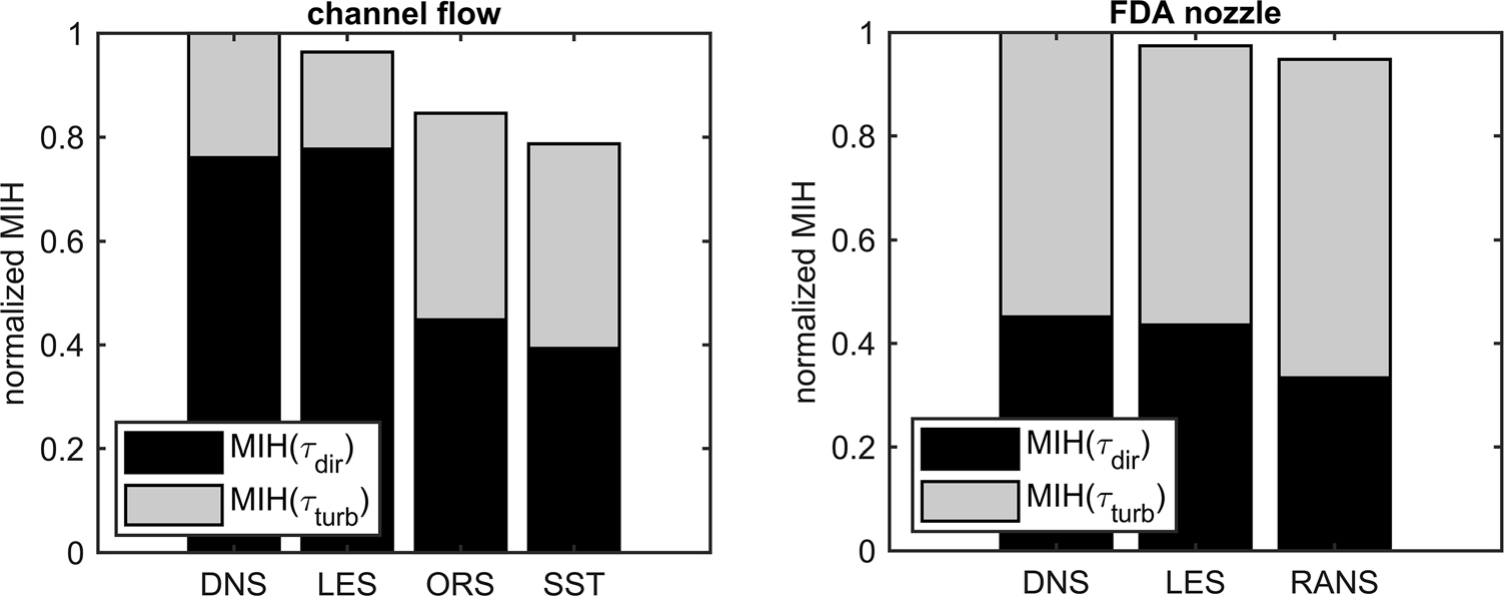
Comparison of calculated sum of $MIH\left( {\tau_{\text{dir}} } \right) + MIH\left( {\langle\tau_{\text{turb}}\rangle } \right)$ and its shares between the used turbulence modelling approaches and DNS for the channel flow and nozzle. The normalized $MIH\left( {\tau_{i} } \right)_{{j,{\text{norm}}}}$ of $j$-th simulation method with $i$-th stress is calculated by $MIH\left( {\tau_{i} } \right)_{{j,{\text{norm}}}} = MIH\left( {\tau_{i} } \right)_{j} \cdot MIH\left( {\tau_{\text{tot}} } \right)_{j} /\left( {MIH\left( {\tau_{\text{tot}} } \right)_{\text{DNS}} \cdot \left( {MIH\left( {\tau_{\text{dir}} } \right)_{j} + MIH\left( {\tau_{\text{turb}} } \right)_{j} } \right)} \right)$ to visualize the shares as a pseudo-sum of mean and turbulent share.

The results are summarized in Fig. 12 for better comparability. All values were normalized for comparability with the respective DNS.

## Discussion

The scalar equivalent stress calculation, including the influence of non-resolved motions, was analyzed and compared between DNS, LES and RANS (two-equation model as well as Reynolds stress model) results. It could be shown that the total stress is in a good agreement, globally and locally, with the baseline from DNS, even if no turbulent movement is resolved by the simulation method (SST, ORS). The most important requirement for turbulence modeling simulations and our stress calculation is that the turbulence model represents the unresolved scales sufficiently accurate. However, this is a general requirement for the use of CFD including turbulence modeling and is not limited to our application. If only mean/direct stresses, i.e. derived from spatial gradients of time-averaged velocities or from RANS results, would be considered for further blood damage prediction as it is done in the state-of-art blood damage modeling, the result are most-likely under-predicted. The comparison of the DNS data to experimental MRI data showed an excellent agreement, which gives confidence in the approach. Even though the resolution of the direct numerical simulations could have been finer in retrospect, i.e. to reduce the ratio between grid resolution and Kolmogorov length scale, and the Courant number, the validation indicates that the turbulent structures were sufficiently resolved to calculate the mean flow correctly.

### Shear Stress Transport

In the core region of the channel flow, the total stress of SST and DNS are nearly similar. Very close to the wall, the model fails to predict the turbulent stresses accurately. This is mainly due to the decrease of modeled turbulent dissipation when approaching the wall. It can be seen in Fig. 8, that the true dissipation of TKE is increasing near the wall. Since the turbulent stress for SST is only influenced by the modeled dissipation, this under-prediction was triggered by the behavior of the SST model. Furthermore, the volumetrically integrated results indicate that the mesh for the RANS calculations was not fine enough to resolve all direct stresses from the mean flow. However, a sufficient near-wall resolution was maintained for the given flow condition. Therefore, it is more likely that the highly active SST model dampens the resolution of direct dissipation. Even if the turbulent share is higher in comparison to DNS and LES, it cannot balance the under-prediction of the direct/mean dissipation to get on the same level as DNS and LES. However, the equivalent scalar stress calculation is clearly improved compared to the standard calculation from the obtained velocity field only without utilizing modeled scales.

The SST model performs well in the fully turbulent region of the nozzle after the sudden expansion. Quite different from the area of the clearly separated shear layer further upstream. Here, the high shear rate in this area causes an increase of eddy viscosity and thus a high modeled dissipation rate, since the shear rate is in the production term of modeled turbulent kinetic energy. As a result, the turbulent stresses, calculated according to our approach, also increase strongly. The increased activity of the turbulence model, in turn, straightens the velocity gradients in the region, which is why the direct/mean mechanical stress is so low in comparison to DNS. In summary, it can be said for the nozzle that the SST model does not reflect the correct influence of turbulent scales to the mean flow under used discretization and boundary conditions. It is known, however, that this model may show weaknesses in case of strong shear layers, transition and especially when both problems are combined. However, the overall stress calculation is improved when modeled turbulent scales are considered. The computed $I\left( {\langle\tau_{\text{tot}}\rangle } \right)$ of the SST model reaches $96 \%$ (channel) and $93 \%$ (nozzle) in comparison to the DNS, but with the use of a discretization with 8 million elements instead of 75 million. The computed $MIH\left( {\langle\tau_{\text{tot}}\rangle } \right)$ of the SST model reaches $79 \%$ (channel) and $95 \%$ (nozzle) in comparison to the DNS, but with the use of a discretization with 8 million elements instead of 75 million.

### Omega Reynolds Stress

Comparable results can be seen between ORS and DNS results. Here, the increase of modeled dissipation near the wall is correctly reflected by the turbulence model. Thus, the total stress and blood damage predicted by ORS is in better agreement with the DNS. It is well-known that Reynolds stress models are superior compared to two-equation models, because they respect the anisotropy of momentum transfer. This is important for wall-bounded flows like in this study. The effect is noticeable in Fig. 8, where the Reynolds stress components are shown in comparison with increasing wall distance. On the other side, Reynolds stress models need more computing time compared to two-equation models, like the used SST closure model. Since the transport of every independent Reynolds stress component is modeled by a transport equation, which leads to six additional differential equations. Thus, an increased computing time by approximately 30% in our case is needed. For our study, this means that the $I\left( {\langle\tau_{\text{tot}}\rangle } \right)$ value is 4 percentage points away from the DNS result and the $MIH\left( {\langle\tau_{\text{tot}}\rangle } \right)$ value is 6 percentage points closer to the DNS result. As already noted for the SST results, the discretization for the resolution of direct stresses does not seem to be sufficiently fine. It can therefore be assumed that the ORS model, with a slight adjustment of the mesh, would further increase its advantage in terms of predicting blood damage.

### Large Eddy Simulation

Again, the total stress is in a good agreement in comparison with the baseline results from DNS. A slight overprediction for the channel flow is noticeable directly near the wall and in the shift between boundary layer and core flow. The overprediction of turbulent kinetic energy is also reflected in the volumetrically integrated stresses in Fig. 11. The main reason should be due to the increased grid resolution between RANS and LES computations. For LES, the near-wall grid in all directions is approximately as fine as for DNS. It was mentioned in the introduction that this is necessary for LES computations. This could lead to already properly reflected turbulent movements in the near-wall region. In combination with a non-zero modeled turbulent dissipation, the total dissipation could be overpredicted in comparison with DNS. Another reason could be an already elsewhere discussed phenomenon of LES turbulence models: In some cases, the turbulent kinetic energy is calculated higher in contrast to the real flow and thus, turbulent dissipation should also be overpredicted.^5^ Nevertheless, the total stress calculated with LES shows the best agreement to the DNS data in the channel. The $I\left( {\langle\tau_{\text{tot}}\rangle } \right)$ reaches $109 \%$ of the value from the DNS of the channel flow, with a mesh of 85 percent fewer elements. This can also be seen in the $MIH\left( {\langle\tau_{\text{tot}}\rangle } \right)$ result, which reaches $96 \%$ and $97 \%$ of the value from the DNS, but with a mesh of 85 percent fewer elements.

The prediction of the flow in the nozzle seems to be more complex for the models used. At both positions the turbulent stresses are partially overestimated by the LES compared to DNS. Although this phenomenon seems to occur in our two cases, it can be said that the total stresses are still better represented when the (overestimated) turbulent components are included rather than when only the mean stresses would be considered for a subsequent blood damage prediction. The most critical point for this problem seems to be the region where the jet breaks down and the majority of the velocity gradients are dominated by turbulent fluctuating movements. The activity of the sub-grid model is basically dependent on the grid size and the resolution of velocity gradients. As a result, it can occur that the grid resolution is not fine enough, and the sub-grid model is not as active as it is supposed to be. As a consequence, the energy budget of the flow is not balanced and this leads to an increase of turbulent kinetic energy at that location and in turn to high turbulent stresses.^5^ The near-wall area at position $Z = 0.024 \,{\text{m}}$ is preferably reflected by LES as with RANS in comparison to DNS. However, the global stress calculation is improved when the unresolved scales are considered. The final $I\left( {\langle\tau_{\text{tot}}\rangle } \right)$ value is only 3 percent points lower in comparison to the baseline DNS.

## Limitations

The conclusions of this study are subject to various limitations and assumptions. The most obvious limitations relate to the calculation of a scalar equivalent stress. Several studies indicate that, for example, red blood cells react differently to normal or shear stresses.^10^,^32^ This study, however, uses an equivalent stress which forms a scalar value from the velocity gradients in each direction. The entire gradient field is considered by this. It is questionable whether, for example, blood cells are located in the near-wall layer where very high velocity gradients are found.^11^ Furthermore, it remains unclear whether large vortices act equally on blood components compared to small turbulent scales. Using dissipation, in particular the modeled dissipation that occurs mainly on the smallest spatial scales and fastest temporal scales, the assumption is added that blood cells react identically to the whole range of turbulent fluctuations. The assumption of a temporally mean flow may be justified regarding the test cases of this study. For blood pumps, which are a globally unstable problem, this assumption is probably not justified and would lead to very rough predictions. Thus, the results of this study are entirely applicable only to locally unstable and incompressible flows of single-phase fluids.

Nevertheless, we consider the results to be significant. With this study we do not want to present an improved blood damage modeling in general, but rather point out that any prediction model could be affected by systematic errors if the turbulent flow field (resolved and unresolved) is not used entirely for the equivalent scalar stress calculation as it is done today.^6^,^9^,^18^,^53^,^60^ This means that the conclusions of this study could be important for the development of new damage models. Only the consideration of all stresses, whether resolved or modeled, can lead to a reliable result in a future prediction model. Especially in the industry efficient methods for the calculation of the flow and the evaluation of the damage potential are used, which model an exceptionally large part of the turbulence and this unresolved share is mostly not considered for the stress calculation and further damage indication. This study firstly addresses to improve the predictive power of such methods so that future patients can benefit from safer devices. And secondly, we want to show the effect of not implementing the modeled part of turbulence in the local and global stress calculation when compared to a direct numerical simulation. However, we are aware that an equivalent scalar stress might be not suitable for a quantitatively correct blood damage prediction. Nevertheless, a scalar equivalent stress is the most widely used approach to date to evaluate the three-dimensional gradient field of a flow in terms of blood damage analysis. Yet, this is not considered relevant because this study was used to compare between different numerical methods and not with experimental blood damage data.

## Conclusion

The target of this study was the examination of whether it is possible to represent the entire mechanical stress field with numerical flow simulations that resolve none or only part of the turbulent share of the flow. The answer to this question is especially important in the field of biomedical flows, because in this discipline very complex and turbulent flow cases (e.g. ventricular assist devices) are simulated with methods that directly resolve either no or only a small part of turbulence of the real flow. However, these turbulences naturally cause fluid-mechanical stresses which are not taken into account with previous methods when predicting potential damage to blood components from CFD. In order to calculate the total, and thus the true stress field according to the chosen boundary conditions and assumptions, direct numerical simulations of two test cases were conducted. First a turbulent channel flow and secondly the flow through the idealized medical device from the FDA “nozzle with sudden expansion”. We validated the results with literature and MRI data. In the DNS results, the whole spectrum of the turbulent fluctuating movements is contained and an equivalent scalar stress was derived with the use of dissipation, which is mathematically synonymous to common notations of equivalent stresses using the second invariant or the norm of the shear rate tensor. Subsequently, the same flows were calculated using closure models and simulation methods, which are of more practical use, and the stress field was derived from this with our scalar equivalent stress, which respects the influence of non-resolved movements. We compared the calculated stresses with those from the baseline DNS locally and globally. This can provide an assessment of the extent to which the prediction of potential blood damage would be altered by using state-of-the-art models and our stress definition. In the case of RANS simulations, the solution field contains no resolved turbulence and the total stresses are determined only by the mean/direct stresses in addition with the portion from the turbulence models, respectively. For LES, on the other hand, there is a resolved share in the turbulent mechanical stresses in addition to the modeled share.

The new scalar equivalent stress, based on the dissipation and with the consideration of unresolved turbulent motions, could be used to calibrate new prediction models. If our hypothesis is correct and the introduced scalar equivalent stress represents all turbulent fluctuations, it should make equivalent scalar calculations out of (U)RANS computations, which are the industrial standard, more reliable. The proposed equivalent stress should have the same level of grid dependency as first-order quantities, i.e. velocity and pressure. This would make different blood damage prediction results out of CFD computations more comparable between each other.

The result was that the total mechanical stresses from RANS and LES computations are in good agreement with the baseline stresses from DNS. The global stress calculation, compared by computing volume integrals over the stresses, is improved by at least 54 percentage points compared between RANS neglecting modeled scales and with modeled scales. The $MIH$ calculation was improved by at least 40 percentage points when the modeled mechanical stresses are taken into account in the prediction and the values from the DNS are used as a basis. From this it can be concluded that this stress definition should be used for numerical simulations of turbulent flows including turbulence modeling, since otherwise gradients that would occur in the fully resolved turbulent flow are simply ignored for the mechanical stress calculation and for further blood damage prediction using RANS or LES. The prerequisite of the proposed scalar equivalent stress is a correct turbulence modeling. The study further indicates and hopefully proves that dissipation is the key parameter when quantifying blood damage within turbulent flows when quantifying blood damage within turbulent flows numerically using an equivalent stress formulation. Only if an adequately experimentally determined prediction model and a numerically obtained stress field, which contains all acting motions, are combined, a generally valid numerical blood damage prediction could become possible in the future. In this context, the new equivalent stress presented in this paper shows how the total equivalent stress can be considered for the application of these damage models to be developed in numerics.
